# ¹⁹F MRI radiomic features: *in vitro* and *in vivo* repeatability

**DOI:** 10.1186/s41747-026-00694-2

**Published:** 2026-03-16

**Authors:** Olga Maxouri, Mariah Daal, Serena Vegna, Diana Ivonne Rodríguez Sánchez, Sajjad Rostami, Stephan Ursprung, Manon Boeije, Natalie Proost, Marieke van de Ven, Leila Akkari, Mangala Srinivas, Zuhir Bodalal, Regina Beets-Tan

**Affiliations:** 1 Department of Radiology, The Netherlands Cancer Institute, Amsterdam, The Netherlands; 2 GROW School for Oncology and Developmental Biology, Maastricht University, Maastricht, The Netherlands; 3 Department of Cell Biology and Immunology, Wageningen University & Research, Wageningen, The Netherlands; 4 Division of Tumor Biology and Immunology, Oncode Institute, The Netherlands Cancer Institute, Amsterdam, The Netherlands; 5 Department of Radiology, Diagnostic and Interventional Radiology, Tuebingen University Hospital, University of Tuebingen, Tuebingen, Germany; 6 Mouse Clinic for Cancer and Aging Research (MCCA), Preclinical Intervention Unit, The Netherlands Cancer Institute, Amsterdam, The Netherlands; 7 The Netherlands Cancer Institute, Amsterdam, The Netherlands; 8 Institute of Regional Health Research, University of Southern Denmark, Odense, Denmark; 9 Maastricht Radiation Oncology, Maastricht, The Netherlands

**Keywords:** Fluorine, Mice, Magnetic resonance imaging, Radiomics, Reproducibility of results

## Abstract

**Objective:**

Using radiomics to compute quantitative imaging features may reveal information beyond standard magnetic resonance imaging (MRI) metrics. We aim to investigate the test-retest repeatability of ¹⁹F MRI radiomic features in phantoms containing two perfluorocarbons and to validate these findings in a pilot *in vivo* mouse tumor model.

**Materials and methods:**

Two phantoms containing perfluoropolyether (PFPE) or perfluoro-15-crown-5 ether (PFCE) were repeatedly scanned (intrasession and intersession) using a 7-T system equipped with a dual-tuned ¹H/¹⁹F volume coil. Radiomic features were extracted and assessed for stability using the concordance correlation coefficient (CCC) ≥ 0.85 and normalized dynamic range ≥ 0.90. A separate *in vivo* test-retest experiment was conducted in tumor-bearing mice injected with a PFPE nanoemulsion.

**Results:**

A total of 194 scans and 772 segments were evaluated across the PFPE phantom, PFCE phantom, and *in vivo* experiments. In both phantoms, radiomic features displayed high intrasession repeatability (median CCC up to 0.886) but decreased intersession repeatability (median CCC down to 0.683). Intensity features were consistently more repeatable (*p* < 0.003) than shape or texture features. We found that 23.1% (466/2,013) of features were repeatable across phantoms. *In vivo* pilot scans showed that 86.1% (401/466) of these phantom-stable features, or ~20.0% overall, remained repeatable under physiological conditions.

**Conclusion:**

Several ¹⁹F MRI-derived features exhibited excellent short-term repeatability, and a considerable proportion proved robust to intersession variability. These robust features may reliably capture ¹⁹F signals under both phantom and physiological conditions, paving the way for more quantitative imaging analysis in this modality and encouraging general reproducibility of data.

**Relevance statement:**

**Key Points:**

- We analyzed 194 ¹⁹F MRI scans and 772 segments obtained in phantoms at 7 T.
- Cross-agent stability identified 466 radiomic features meeting concordance correlation coefficient ≥ 0.85 and normalized dynamic range ≥ 0.90.
- Of these phantom-stable features, 401 of 466 remained stable *in vivo* in a tumor mouse model.
- Intensity features were most repeatable, while shape features were least stable across sessions.
- Median concordance correlation coefficient dropped from 0.886 intrasession to 0.683 intersession.

**Graphical Abstract:**

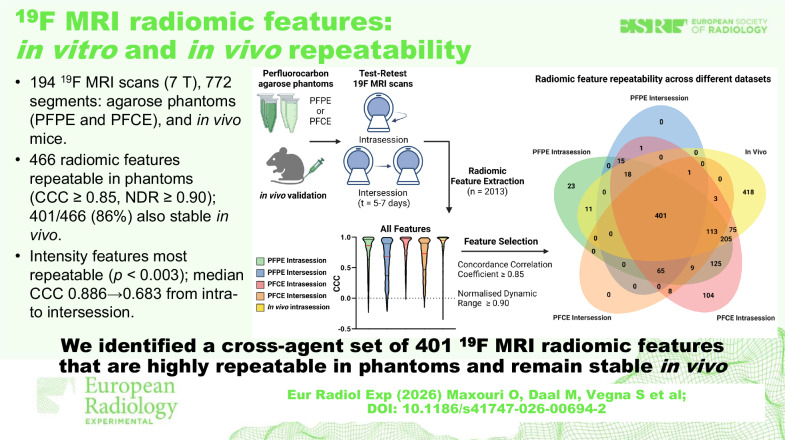

## Background

Fluorine-19 (¹⁹F) magnetic resonance imaging (MRI) has emerged over the past two decades as a promising modality for cellular and molecular imaging [1–5]. Its unique advantages, background-free imaging, the absence of ionizing radiation, the ability for absolute quantification, and the capacity for multispectral imaging, make it ideal for cell tracking (*e.g*., for immunotherapy) and targeted imaging of inflammatory processes [6–8]. Emerging human studies explore the feasibility of ¹⁹F MRI toward clinical application [9–11].

A unique characteristic of ¹⁹F MRI is its inherent suitability for quantitative analyses, as the signal intensity is directly proportional to the concentration of ¹⁹F nuclei [12]. This linear relationship helps address known limitations associated with contrast agents in conventional proton (¹H) MRI, such as non-specific signal enhancement and difficulties in quantification [13]. Traditional quantitative approaches in ¹⁹F MRI typically focus on single metrics, such as spin density or longitudinal relaxation rates, to quantify ¹⁹F-labeled cells and oxygen tension [14, 15]. However, relying solely on single metrics for quantification may overlook additional information crucial for understanding complex biological processes. Expanding to imaging markers and their dynamics in ¹⁹F and ¹H MRI may reveal meaningful imaging phenotypes.

Radiomics offers a promising solution by objectively extracting many quantitative features, termed radiomic features, from medical images for comprehensive data analysis. In oncology, radiomics has demonstrated effectiveness in improving prognosis and treatment predictions by capturing tumor heterogeneity and subtle imaging characteristics [16, 17]. This is represented by the extracted radiomic features, which include intensity, size, shape, texture, and spatial relationships of structures within the image, revealing details far beyond what the naked eye can perceive [18, 19]. Radiomics has been used to predict prognosis [20], response to cancer treatment [21], the emergence of adverse clinical events [22], and underlying tumor biology [23, 24]. Applying radiomics to ¹⁹F MRI could unlock new insights into cellular and molecular dynamics by capturing a broader spectrum of quantitative information.

Before (pre-)clinical implementation, evaluating the reliability, specifically repeatability, of radiomic features extracted from ¹⁹F MRI is essential to ensure that features are robust and generalizable across studies [25, 26]. Factors such as equipment differences, imaging parameters, and image processing algorithms can influence these features [16]. Previous studies have assessed the robustness of imaging markers in various imaging modalities [27–29], but to our knowledge, radiomics analysis of ¹⁹F MRI has not yet been explored.

This study aims to fill this critical gap by investigating the repeatability of radiomic features extracted from ¹⁹F MRI in test-retest scans of an agarose phantom containing perfluorocarbon polyfluoropolyether (PFPE). We further validate the repeatability of these features *in vitro* using test-retest scans of a phantom containing another perfluorocarbon, perfluoro-15-crown-5 ether (PFCE), and *in vivo* experiments in a heterotopic tumor mouse model.

## Materials and methods

Ethical approval was waived for the phantom experiments, as they did not involve human or live animal subjects. The *in vivo* validation study received approval from the Institutional Animal Ethics Committee and complied with the relevant guidelines (17.1.10220, AVD301002017378).

### Phantom design

Two agarose phantom setups were constructed to evaluate the repeatability of radiomic features in ¹⁹F MRI. The first phantom consisted of five 0.5-mL microcentrifuge tubes arranged in a custom three-dimensional (3D)-printed holder, maximizing the number of regions of interest (ROIs) within a scan. Four tubes were filled with a mixture of phosphate-buffered saline and 1% (w/v) low-melting-point agarose (UltraPure Low Melting Point Agarose, Invitrogen), along with a commercially available PFPE nanoemulsion (VS-1000H DM Red, Celsense, mean droplet size ~145 nm) simulating multiple ¹⁹F “hot spots.” The use of an agarose hydrogel instead of a solution facilitates even dispersion of the nanoemulsion and mimics the physical environment surrounding the imaging agent *in vivo*. The fifth tube contained agarose without PFPE as a control.

To capture signal heterogeneity relevant for intensity- and texture-based features, different PFPE concentrations were used between ROIs, approximating signal intensities observed at sites of inflammation. Two tubes contained PFPE at 5.8 mM (corresponding to a ¹⁹F nuclei content of 244 mM), and two contained 2.8 mM PFPE (118 mM ¹⁹F nuclei content). These concentrations are above our system’s detection limit (Supplementary Fig. S1) while avoiding signal spillover at high fluorine concentrations (*e.g*., 400 mM) or loss at low concentrations (*e.g*., 50 mM). A PFCE concentration series was acquired to establish a practical detection floor for the ¹⁹F MRI protocol. PFCE was chosen for this calibration because its single ¹⁹F resonance provides a straightforward relationship between total signal intensity and fluorine concentration, enabling accurate determination of sensitivity limits. Because spin-density–weighted ¹⁹F signal scales with the number of ¹⁹F nuclei, a single-agent calibration was sufficient to set phantom concentrations above the sensitivity threshold for all experiments. This configuration allowed robust delineation of object boundaries (signal-to-noise ratio (SNR) > 5), enabling the evaluation of shape feature repeatability under consistent geometric conditions.

The second phantom, used for *in vitro* validation, contained four tubes filled with phosphate-buffered saline and 1% (w/v) low-melting-point agarose. Poly (lactic-co-glycolic acid) nanoparticles loaded with PFCE (20 ^19^F nuclei/molecule) at matching ¹⁹F concentrations to the first phantom (~42 ^19^F nuclei/molecule) were used for comparison. Previous literature describes the synthesis and characterization of these nanoparticles in detail [30]. The PFCE nanoparticle batch used in the phantom had an average size of 167 nm and a polydispersity index of 0.079.

We selected PFPE and PFCE as representative ¹⁹F tracers because they cover the spectral and relaxation behavior commonly seen in ¹⁹F MRI. PFCE has a single narrow resonance that simplifies frequency selection and suppresses chemical-shift–related artifacts. In contrast, PFPE offers a very high ¹⁹F payload per molecule but a multi-peak spectrum. Both agents are widely used for cell tracking and imaging inflammation in preclinical studies. Phantoms were stored at 4 °C between imaging sessions to preserve gel structure and nanoemulsion/nanoparticle stability.

### Animal husbandry, cell line, and *in vivo* model

An *in vivo* study used tumor-bearing mice (*n* = 2) to confirm the phantom findings under physiological conditions. The *in vivo* analysis serves as a pilot confirmation of the phantom-derived core and is hypothesis-generating rather than powered for clinical generalization. Supplementary Methods Section 1 details animal handling and cell lines used to generate the *in vivo* tumor model. We selected a specific heterotopic hepatocellular carcinoma model because it has been shown to robustly recruit tumor-associated macrophages [31–33], phagocytic cells that internalize perfluorocarbon nanoemulsions and generate a detectable ¹⁹F signal. Tumor-bearing mice received intravenous injections of PFPE nanoemulsion (200 μL, 120 mg PFPE/mL) and were scanned twice (test-retest) 2 days after injection, consistent with the approach used for the phantoms. For the pilot *in vivo* validation, we used PFPE only, as dosing, safety, and ethical approvals were already established for this agent, and a GMP-compatible research formulation is commercially available. Restricting the *in vivo* arm to a single agent reduced the number of animals (in line with the 3Rs principles [34]) while still testing whether phantom-stable features remain robust under physiological conditions.

### MRI protocol

Imaging was conducted on a 7-T BioSpec 70/20 USR scanner equipped with a 40-mm-diameter dual-tuned ¹H/¹⁹F transmit-receive volume coil (Bruker), which was tuned and matched before each session. Phantoms and mice were consistently positioned in the coil, using the same geometry whenever possible. Before ¹⁹F image acquisition, the resonant frequency of the perfluorocarbon contrast agents was determined using a nonlocalized free induction decay ¹⁹F MRS sequence with an acquisition bandwidth of 50 kHz, repetition time = 2,000 ms, and the number of excitations = 1‒10. The resulting spectrum was analyzed using TopSpin (ParaVision 6.0.1/7.0.1, Bruker).

Structural ¹H images were acquired with a turbo rapid acquisition with relaxation enhancement (RARE) sequence (repetition time/echo time = 2,500/36 ms, turbo factor = 8, FOV = 35 × 35 mm², acquisition matrix = 256 × 256, slice thickness = 1.5 mm, bandwidth = 72 kHz, number of excitations = 2; acquisition time = 2:40 min:s) and used to define the geometry for ¹⁹F imaging. Matched ¹⁹F turbo RARE imaging used a similar geometry (repetition time/echo time = 2,000/22.5 ms, turbo factor = 8, FOV = 35 × 35 mm², acquisition matrix = 64 × 64, slice thickness = 1.5 mm, bandwidth = 20 kHz, number of excitations = 120; acquisition time = 32 min). The voxel size was 0.55 × 0.55 × 1.5 mm³. All parameters were kept constant across phantom and *in vivo* scans. Detailed image acquisition parameters are provided in Supplementary Table S1. Noise scans were acquired and analyzed slice-by-slice to estimate noise levels for subsequent image processing.

For the *in vivo* validation, mice were anaesthetized with isoflurane (1–2%), and respiration was monitored using a respiratory pillow sensor. Respiratory gating was used to minimize motion artifacts. Body temperature was maintained with a warm water-circulating heating pad. A reference tube containing 5 mM PFPE was placed alongside the animal in the coil. Two days after the PFPE injection, a scout scan was performed for positioning and slice selection, followed by ¹H/¹⁹F MRI (Supplementary Fig. S2).

### Data preparation and segmentation

To distinguish true ¹⁹F signal from noise, a threshold of three times the average noise level (N_average_) was applied, exceeding five times the noise’s standard deviation. Pure noise images were acquired to determine N_average_, calculated on a slice-by-slice basis [35]. Images were exported in Digital Imaging and Communications in Medicine‒DICOM format, processed, and segmented using 3D Slicer (v.5.2.2). Semi-automatic segmentation proceeded in two steps: (1) a mask was generated including only voxels with intensity ≥ three times the N_average_; (2) the region of interest for each phantom tube or anatomical structure (*in vivo*) was manually delineated (within the thresholded mask) by a blinded observer. In phantoms, each tube was defined as a region of interest; *in vivo*, only the tumor, liver, and reference tube were outlined using both ¹H and ¹⁹F images for anatomic guidance. This prevented the possible inclusion of isoflurane artifact-influenced voxels in our analyses.

### Feature extraction

Radiomic features were extracted using PyRadiomics v3.1.0a2 [36]. A total of 2,016 features were calculated, encompassing first-order statistics (intensity), shape features, and texture-based features: gray level co-occurrence matrix features, gray level dependence matrix features, gray level run length matrix features, gray level size zone matrix features, and neighboring gray tone difference matrix. Routine quantitative variables used in ¹⁹F MRI assessment, such as mean signal intensity, total energy (integrated signal), and ROI volume or sphericity, are directly represented within these first-order and shape feature families and are therefore included in our repeatability analyses. Filters applied included ‘Original’ (*i.e*., no filter), Square, Square-root, Logarithm, Exponential, Gradient, Laplacian of Gaussian, Wavelet, and Local binary pattern. Extraction settings are described in Supplementary Methods Section 2. Three radiomic features were excluded due to technical reasons (Supplementary Methods Section 3), resulting in a final pool of 2,013 features for the analyses. Detailed feature descriptions are available in van Griethuysen et al [36].

### Study design

**Fig. 1:**
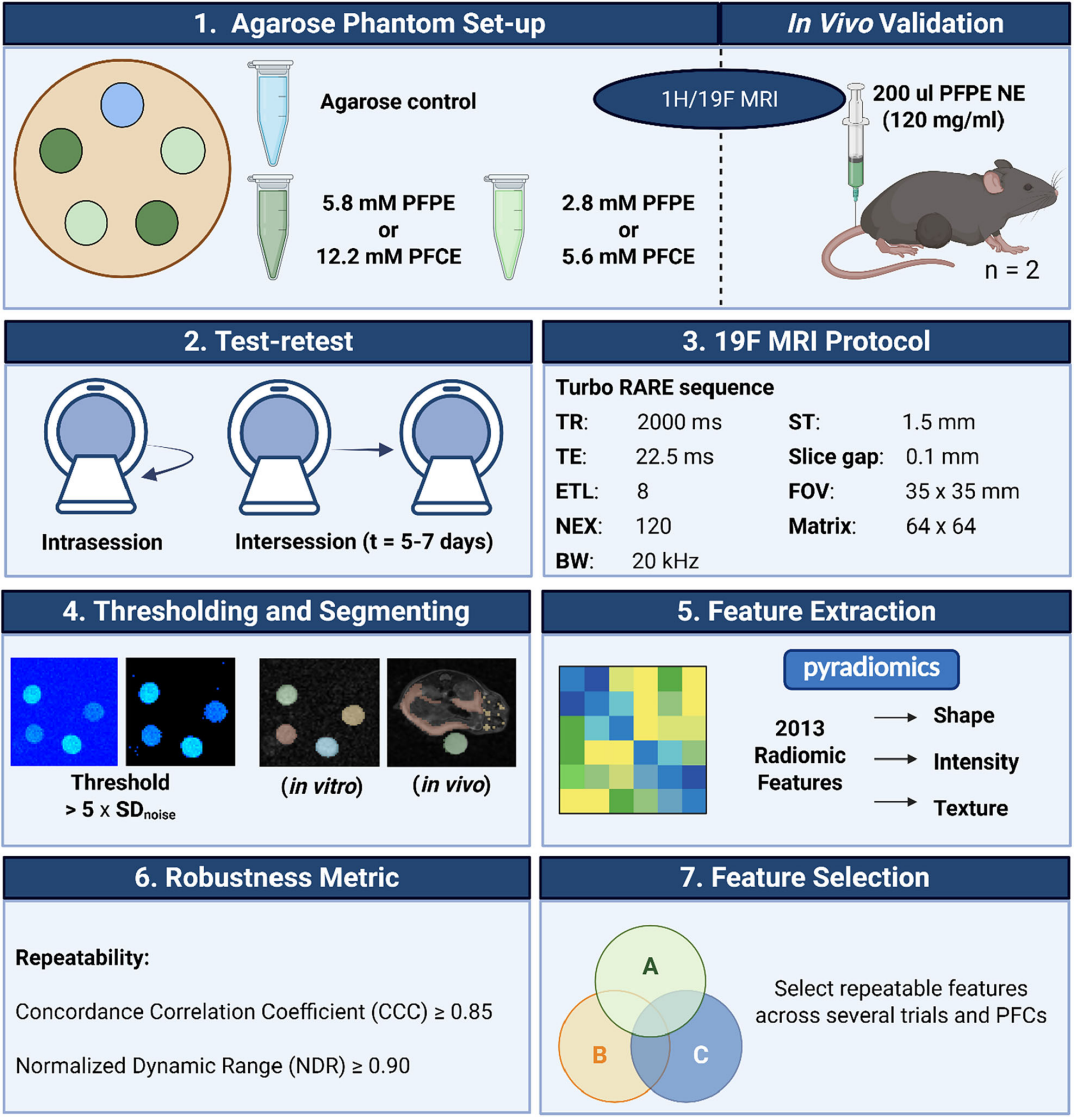
Study design for assessing repeatability of radiomic features in ¹⁹F MRI. (1.) Phantoms containing tubes filled with agarose and perfluorocarbons (PFPE or PFCE) were imaged using ¹H MRI to locate the tubes and guide subsequent ¹⁹F MRI scans. (2.) Two test-retest trials were conducted: (i) intrasession scans performed sequentially on the same day without repositioning (applied to both phantoms and *in vivo* studies), and (ii) intersession scans performed 5–7 days apart with complete repositioning (applied to phantoms only). (3.) Scans were acquired using a fast spin-echo (TurboRARE) sequence. (4.) Semi-automatic segmentation was performed by applying an image mask at SNR > 5 and manual delineation of the ROIs. (5.) Radiomic features were extracted from segmented regions of interest in the ¹⁹F MRI scans to assess repeatability. (6.) Repeatable features were selected using the concordance correlation coefficient, and the normalized dynamic range was used additionally to identify stable features. (7.) Features that made the thresholds were subsequently compared among different trials, assessing the degree of overlap between the groups. BW, Bandwidth; ETL, Echo train length; FOV, Field of view; NEX, Number of excitations; PFCE, Perfluoro-15-crown-5 ether; PFPE, Perfluoropolyether; ROI, Region of interest; SNR, Signal-to-noise ratio; ST, Slice thickness; TE, Echo time; TR, Repetition time

**Fig. 2:**
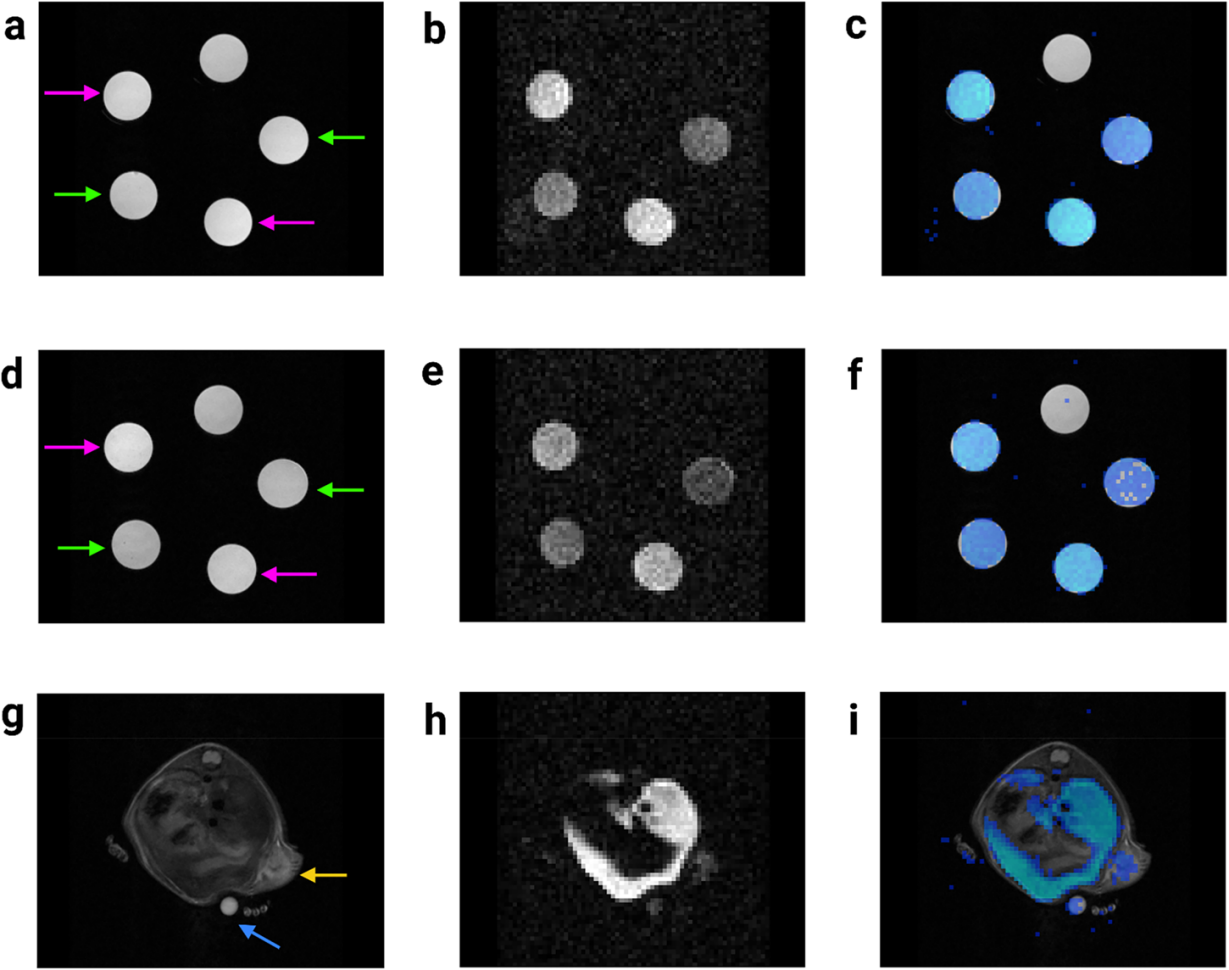
Representative ¹H/¹⁹F MRI scans of the PFPE and PFCE phantoms, and *in vivo* imaging with intravenously injected PFPE nanoemulsion. **a** Axial ¹H MRI of the PFPE phantom showing five 0.5 mL microcentrifuge tubes filled with agarose mixtures containing PFPE at concentrations of 5.8 mM (magenta arrows) and 2.8 mM (green arrows); the fifth tube is a negative control without PFPE. **b** Corresponding ¹⁹F MRI of the PFPE phantom. **c** Overlay of ¹H and ¹⁹F MRI scans for anatomical reference, with ¹⁹F threshold set at a signal-to-noise ratio > 3. **d** Similar ¹H imaging of the PFCE phantom with PFCE concentrations of 12.2 mM (magenta arrows) and 5.6 mM (green arrows). **e** Corresponding ¹⁹F MRI of the PFCE phantom. **f** Merged ¹H/¹⁹F image of the PFCE phantom with thresholding. **g** *In vivo* axial ¹H MRI of a mouse abdomen depicting the liver, subcutaneous tumor (yellow arrow), and a reference tube containing 5 mM PFPE (blue arrow). **h** Corresponding ¹⁹F MRI showing PFPE accumulation *in vivo*. **i** Overlay of ¹H and ¹⁹F MRI scans highlighting the anatomical location of PFPE accumulation at the liver and tumor with superimposed and thresholded ¹⁹F signal. PFCE, Perfluoro-15-crown-5 ether; PFPE, Perfluoropolyether

In this prospective study, we conducted experiments using different subjects and test-retest trials to evaluate the repeatability of features extracted from ¹⁹F MRI images (Fig. 1). Two agarose phantoms were created, each comprising a homogenous mixture of agarose with a commonly used perfluorocarbon (PFPE or PFCE) to assess robust features independent of the specific imaging/contrast agent. Additionally, a pilot *in vivo* experiment using a heterotopic hepatocellular carcinoma mouse model (*n* = 2) was performed to validate the findings under physiological conditions. The phantoms and the murine model underwent ¹⁹F MRI acquisition twice within a single scanning session without repositioning (intrasession trial) to assess repeatability under consistent conditions. To evaluate reproducibility over time and with repositioning, an intersession trial was conducted by performing additional scans on both phantoms 5–7 days after the initial imaging session. *In vivo* repeatability was assessed intrasession only to minimize anesthesia time and handling (3Rs) and to avoid biological confounders between days (*e.g*., nanoparticle redistribution/clearance and tumor growth) that could obscure pure repeatability analysis. Multiday intersession effects were therefore comprehensively quantified in phantoms where geometry and composition are stable. Each scan underwent threshold-based semi-automatic segmentation before radiomic feature extraction (Fig. 2).

In this study, we use the term ‘scan’ to denote a single ¹⁹F MRI acquisition of either a phantom or a mouse at a given time point. Within each scan, individual ROIs were delineated and are referred to as segments. For the phantoms, each scan contained four perfluorocarbon-loaded tubes, each segmented as a separate ROI (four segments per phantom scan). For the *in vivo* experiments, each scan included three segments: the tumor, the liver, and a ¹⁹F reference tube.

Repeatable features were identified using the statistical methods described below. The intersection of these features across the different experiments was further examined to identify robust radiomic features applicable across various settings. This work followed the 2024 CLAIM checklist to ensure comprehensive and transparent reporting of imaging-based artificial intelligence research [37].

### Statistical analysis

The repeatability of radiomic features was evaluated using the concordance correlation coefficient (CCC), which measures the agreement between test and retest measurements [38]:

1 $${CCC}=\frac{2{\sigma }_{12}}{{\sigma }_{1}^{2}+{\sigma }_{2}^{2}+{({\mu }_{1}-{\mu }_{2})}^{2}}\,$$

where ${\sigma }_{12}$ is the covariance between test and retest measurements, ${\sigma }_{1}^{2}$ and ${\sigma }_{2}^{2}$ are the variances, and μ_1_ and μ_2_ are the means of the test and retest, respectively. A CCC value of 1 indicates perfect positive agreement, while a value of -1 indicates perfect negative agreement. In line with previous feature robustness studies, a cut-off of 0.85 was used to designate high repeatability [39]. The normality of CCC distributions was assessed using the Shapiro–Wilk test; based on these results, non-parametric tests were used for all subsequent analyses (Supplementary Table S2). The Kruskal–Wallis test was used to compare distributions of CCC across groups, with post hoc multiple testing correction applied using the Benjamini–Hochberg method. Bland–Altman plots were computed with 95% limits of agreement and a reference band at expected CCC = 0.85 as described by Kim and Lee [40].

To assess the informativeness of a radiomic feature, the normalized dynamic range (NDR) was calculated for the different test-retest settings [27]:

2 $${{DR}}_{k}=\left(1-\frac{1}{n}{\sum }_{i=1}^{\eta }\frac{|{{Test}}_{k}(i)-{{Retest}}_{k}(i)|}{{{Max}}_{k}-\,{{Min}}_{k}}\right)$$

where *n* is the number of observations, and Max_k_ and Min_k_ are the maximum and minimum values of feature k across all observations. As Balagurunathan et al recommended [27], we used an NDR threshold of ≥ 0.90 to ensure that each feature possessed sufficient dynamic range. We further combined this criterion with a CCC ≥ 0.85, designating features that met both thresholds as “stable.”

To assess segmentation reproducibility, we evaluated both intra- and inter-reader variability on ¹⁹F MRI phantom scans using the same semi-automatic segmentation protocol described in the main Methods. Intra-reader variability was assessed by repeating segmentations on 20 PFPE scans by a single observer, while inter-reader variability was evaluated across two independent readers on 40 scans (20 PFPE and 20 PFCE). For each feature, ICC values were computed using the *pingouin* package. Further details are provided in Supplementary Methods Section 4.

Statistical analyses were performed using Python (v3.10.6) and *pandas v2.2.3*, *numpy v2.2.1*, *scipy v1.11.1*, *statsmodels v0.14.4*, *nrrd v0.4.3*, *matplotlib v3.10.0*, *pyradiomics v3.1.0a2*, *trimesh v4.0.5*, *pingouin v0.5.5*.

## Results

A total of 194 scans and 772 segments were evaluated across the PFPE phantom, PFCE phantom, and *in vivo* experiments (Table 1). In the PFPE phantom experiments, each of the 54 scans contained four perfluorocarbon-loaded tubes, resulting in a total of 216 segments. Similarly, the PFCE phantom experiments involved 544 segments from 136 scans. For *in vivo* validation, two mice underwent an intrasession test-retest ¹⁹F MRI, resulting in a total of four scans. Each scan contained three regions of interest: the liver, the tumor, and a ¹⁹F reference tube, for a total of 12 segments. Per-ROI voxel counts are reported in Supplementary Table S3.

**Table 1:** Summary of scans and segments (regions of interest) in the perfluoropolyether (PFPE) phantom, the perfluoro-15-crown-5-ether (PFCE) phantom, and *in vivo* studies

Phantom/study type	Intrasession test-retest scans	Intrasession test-retest segments	Intersession test-retest scans	Intersession test-retest segments	Total scans	Total segments
PFPE Phantom	18	72	36	144	54	216
PFCE Phantom	68	272	68	272	136	544
*In vivo* validation	4	12	–	–	4	12

Subsequently, a blinded observer delineated each test and retest scan using a two-step semi-automatic approach that aimed to minimize observer-dependent variability, as reflected in the high intra-reader stability of the resulting features (median ICC = 0.99978, interquartile range (IQR) = 0.99906–0.99994). A second observer blindly delineated a subset of scans using the same semi-automatic approach to assess interobserver variability (median ICC = 0.99997, IQR = 0.99977–0.99999, Supplementary Methods Section 4).

**Fig. 3:**
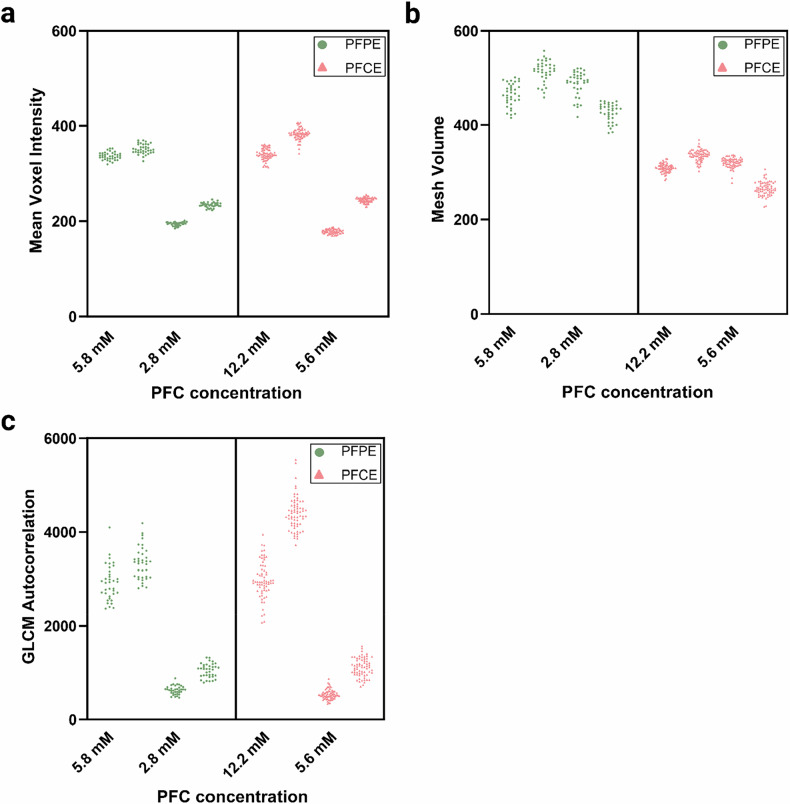
Comparison of ¹⁹F radiomic features between PFPE and PFCE phantoms. Scatter plots illustrating examples of radiomic features extracted from individual microcentrifuge tubes in the PFPE (green circles) and PFCE (red triangles) phantoms at different concentrations. **a** First-order feature example: mean voxel intensity. **b** Shape feature example: mesh volume. **c** Texture feature example: gray level co-occurrence matrix (GLCM) autocorrelation. Radiomic features were extracted from regions of interest in ¹⁹F MRI scans using a turbo RARE sequence. CCC, Concordance correlation coefficient; PFC, Perfluorocarbon; PFCE, Perfluoro-15-crown-5 ether; PFPE, Perfluoropolyether

Representative scatter plots of intensity, shape, and texture features from individual microcentrifuge tubes in both phantoms are shown in Fig. 3a–c. Intensity normalization was also assessed in a representative scan set (Supplementary Figs. S3 and S4). As the differences in test-retest intensity histogram similarity between non-normalized and normalized images were minimal (mean Δ% Jensen–Shannon divergence = 0.7% for intrasession, 5.2% for intersession), only the original images were used in further analyses.

### PFPE phantom results

To assess the robustness of features in PFPE-labeled samples, we first examined test-retest scans acquired consecutively (intrasession). Overall, PFPE-derived radiomic features demonstrated high intrasession repeatability (median overall CCC = 0.859, IQR = 0.633–0.965). Stratification by feature class (*i.e*., shape, intensity, and texture) showed that intensity features had the highest intrasession repeatability (median CCC = 0.917, IQR = 0.783–0.985), significantly exceeding the overall intrasession CCC (*p* < 0.001). Shape features (median CCC = 0.842, IQR = 0.739–0.876) were not more robust than overall (*p* = 0.499), whereas texture features (median CCC = 0.846, IQR = 0.606–0.958) were modestly but significantly less repeatable (*p* = 0.047). Overall, the Laplacian of Gaussian, wavelet low-low-low, square, and exponential filters yielded more repeatable features in the PFPE intrasession setting (Supplementary Fig. S5).

In the intersession setting, where retest scans were acquired 5–7 days later to avoid sedimentation artifacts (Supplementary Methods Section 5, Supplementary Fig. S6), the overall feature robustness decreased significantly (median CCC = 0.683, IQR = 0.370–0.891, *p* < 0.001). Intensity features again showed the highest repeatability (median CCC = 0.722, IQR = 0.419–0.940, *p* = 0.003), whereas shape features (median CCC = 0.558, IQR = 0.010–0.647) were significantly lower than overall (*p* = 0.013). Texture features (median CCC = 0.669, IQR = 0.359–0.879) were comparable to the overall CCC (*p* = 0.305). All feature classes exhibited significantly reduced repeatability in the intersession setting compared to the intrasession setting (*p* < 0.001).

### PFCE phantom results

Similar to the PFPE phantom, the PFCE phantom demonstrated strong intrasession reliability, with a median overall CCC = 0.886 (IQR = 0.692–0.973). Among feature classes, intensity features again achieved the highest intrasession repeatability (median CCC = 0.958, IQR = 0.835–0.989), surpassing the overall CCC (*p* < 0.001). Shape features (median CCC = 0.891, IQR = 0.854–0.938) did not differ significantly from the overall CCC (*p* = 0.826), while texture features (median CCC = 0.868, IQR = 0.656–0.967) were moderately higher than overall (*p* = 0.007).

**Fig. 4:**
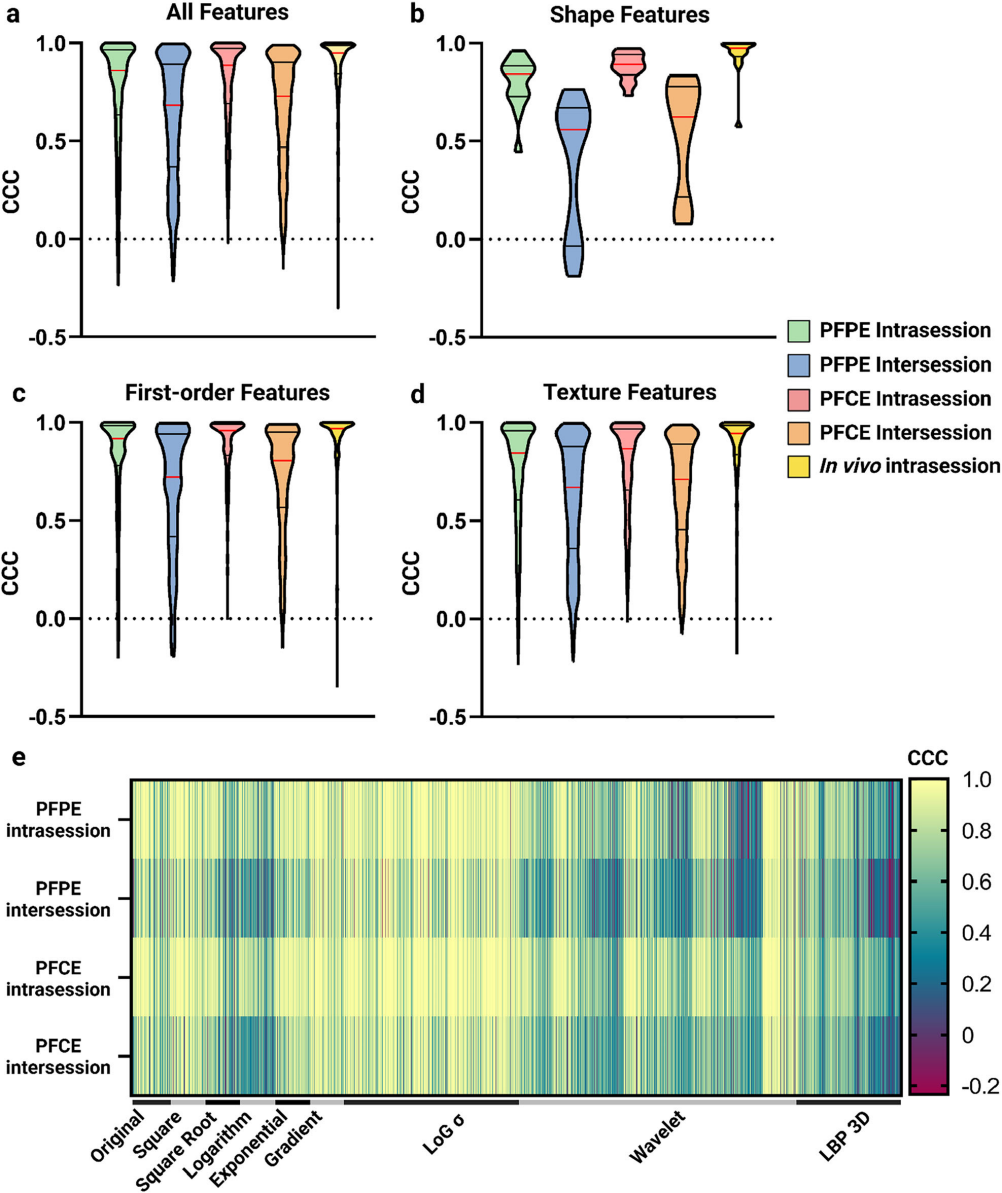
Distribution of CCC values and per feature class across test-retest trials. Violin plots representing the CCC distributions in various test-retest trials for different radiomic feature classes: (**a**) all features (*n* = 2,013); (**b**) shape features (*n* = 14); (**c**) first-order features (*n* = 394); and (**d**) texture features (*n* = 1,605). The median (red line) and quartiles (black lines) are depicted for each trial. **e** Heatmap displaying the CCC of the 2,013 extracted features, arranged per filter, in different test-retest trials and phantoms, indicating similar repeatability patterns between phantoms and reduced repeatability in intersession trials. CCC, Concordance correlation coefficient; PFCE, Perfluoro-15-crown-5 ether; PFPE, Perfluoropolyether

In the intersession setting, feature robustness significantly decreased (median CCC = 0.729, IQR = 0.468–0.902, *p* < 0.001). Intensity features again exhibited the highest repeatability (median CCC = 0.807, IQR = 0.567–0.950) and differed significantly from the overall CCC (*p* < 0.001). Shape (median CCC = 0.623, IQR = 0.285–0.772, *p* = 0.086) and texture (median CCC = 0.711, IQR = 0.456–0.891, *p* = 0.144) did not differ significantly from the overall CCC. As with the PFPE phantom, all PFCE feature classes showed markedly lower repeatability intersession than intrasession (*p* < 0.001), highlighting that repositioning or day-to-day variations can affect feature reliability (Table 2, Fig. 4a–d). This trend is further illustrated in Bland–Altman plots of individual radiomic features (Supplementary Fig. S7), depicting decreased agreement in the intersession test-retest trial.

**Table 2:** Repeatability of radiomic features (median concordance correlation coefficient, interquartile range) across different categories (overall, shape, intensity, and texture) for the perfluoropolyether (PFPE) phantom and the perfluoro-15-crown-5-ether (PFCE) phantom in intrasession and intersession settings

	Overall features	Shape features	Intensity features	Texture features
PFPE intrasession	0.859 (0.633–0.965)	0.842 (0.739–0.876)	0.917*** (0.783–0.985)	0.846* (0.606–0.958)
PFPE intersession	0.683 (0.370–0.891)	0.558* (0.010–0.647)	0.722** (0.419–0.940)	0.669 (0.359–0.879)
PFCE intrasession	0.886 (0.692–0.973)	0.891 (0.854–0.938)	0.958*** (0.835–0.989)	0.868** (0.656–0.967)
PFCE intersession	0.729 (0.468–0.902)	0.623 (0.285–0.772)	0.807*** (0.567–0.950)	0.711 (0.456–0.891)

The stars indicate the significance levels of the difference between each of the feature classes (shape, intensity, texture) and their respective overall features’ concordance correlation coefficients, where * corresponds to an adjusted *p*-value < 0.05, ** corresponds to an adjusted *p*-value < 0.01, and *** corresponds to an adjusted *p*-value < 0.001

Direct comparisons of PFPE and PFCE revealed that PFCE generally showed higher intrasession repeatability for overall (*p* < 0.001), intensity (*p* = 0.002), and texture features (*p* < 0.001). In contrast, shape features did not vary significantly between the two contrast agents (*p* = 0.072). In the intersession setting, PFCE outperformed PFPE in overall and texture feature repeatability (*p* < 0.001), whereas shape (*p* = 0.108) and intensity features (*p* = 0.081) were comparable. Figure 4e presents a heatmap of CCC values for radiomic features grouped by filter, showing high intrasession and lower intersession repeatability patterns observed with both perfluorocarbon contrast agents.

### Feature stability

**Fig. 5:**
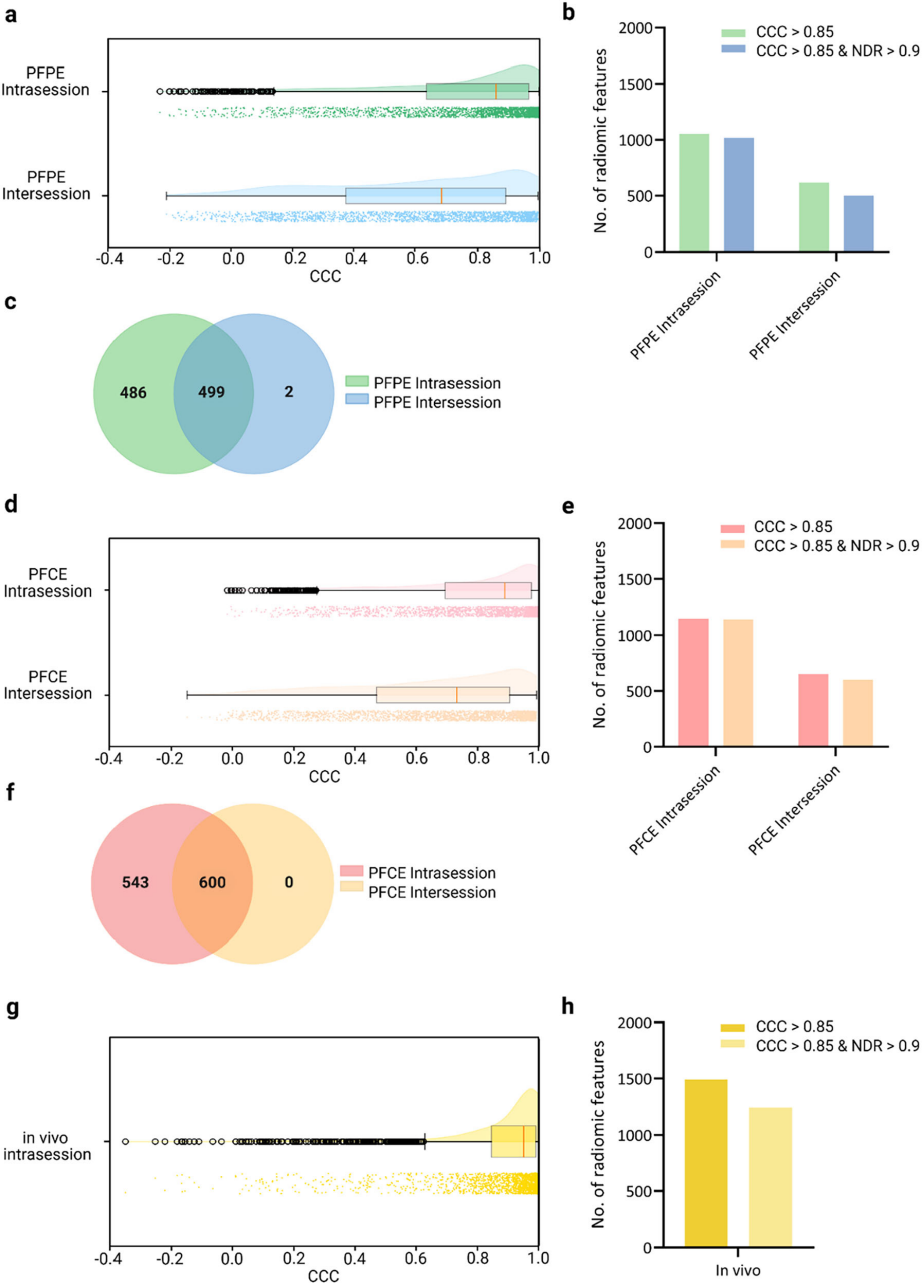
Repeatability of ¹⁹F radiomic features in intrasession and intersession test-retest trials. Raincloud plots showing the distribution of CCC values for radiomic features extracted from ¹⁹F MRI scans of (**a**) the agarose PFPE phantom, (**d**) the agarose PFCE phantom, and (**g**) the *in vivo* model. Each plot includes a half-violin diagram illustrating data density, a box plot indicating the median and interquartile ranges, and individual data points representing the CCC values of the features. **b**, **e**, **h** Bar graphs depicting the number of repeatable radiomic features (CCC ≥ 0.85) in intra- and intersession trials, with and without applying the normalized dynamic range (NDR) threshold. **c**, **f** Venn diagrams showing the overlap of stable radiomic features (meeting both CCC and NDR thresholds) between intrasession and intersession trials in the PFPE and PFCE phantoms, respectively. CCC, Concordance correlation coefficient; PFCE, Perfluoro-15-crown-5 ether; PFPE, Perfluoropolyether

To identify a subset of stable radiomic features, we applied a combined threshold of CCC ≥ 0.85 and NDR ≥ 0.90 to all radiomic features in each experiment (Table 3). In the PFPE phantom, 48.9% (985/2,013) of features were stable in the intrasession setting, with intensity metrics showing the highest proportion (62.9%, 248/394 intensity features), followed by shape (50.0%, 7/14 shape features) and texture (45.5%, 730/1,605 texture features). However, when the intersession setting was assessed, overall stability fell to 24.9% (501/2,013). Shape features were particularly sensitive to these longer intervals (0% stability), whereas some intensity (31.0%, 122/394) and texture (23.6%, 379/1,605) features remained repeatable. Overall, 24.8% (500/2,013) of PFPE features satisfied the stability criteria across both intrasession and intersession analyses (Fig. 5a–c).

**Table 3:** The number of stable 19 F MRI radiomic features identified in the perfluoropolyether (PFPE) phantom, the perfluoro-15-crown-5-ether (PFCE) phantom, as well as in an *in vivo* setting (*n*, % of that feature type)

	Stable radiomic features	Total radiomic features	Stable intensity features	Total intensity features	Stable shape features	Total shape features	Stable texture features	Total texture features
PFPE intrasession	1,017 (50.5%)	2,013	255 (64.7%)	394	7 (50.0%)	14	755 (47.0%)	1,605
PFPE intersession	501 (24.9%)	2,013	122 (31.0%)	394	0 (0.0%)	14	379 (23.6%)	1,605
PFCE intrasession	1,143 (56.8%)	2,013	282 (71.6%)	394	11 (78.6%)	14	850 (53.0%)	1,605
PFCE intersession	600 (29.8%)	2,013	145 (36.8%)	394	0 (0.0%)	14	455 (28.3%)	1,605
*In vivo* intrasession	1,245 (61.8%)	2,013	264 (67.0%)	394	11 (78.6%)	14	970 (60.4%)	1,605

Stable features are defined as having a concordance correlation coefficient ≥ 0.85 and a normalized dynamic range ≥ 0.90

A similar trend was observed with PFCE, although intrasession stability was higher at 56.8% (1,143/2,013). Shape features showed the greatest intrasession robustness (78.6%, 11/14), surpassing both intensity (71.6%, 282/394) and texture (53.0%, 850/1,605). Again, none of the shape features remained stable in the intersession test-retest, and only a minority of intensity (36.8%, 145/394) and texture (28.3%, 455/1,605) features remained consistently stable between scans. Despite this decline, 29.8% (600/2,013) of features fulfilled the stability criteria in both intrasession and intersession scans for the PFCE phantom (Fig. 5d–f).

When comparing these stable sets across the two phantoms, 23.1% of all radiomic features (466/2,013) fulfilled the CCC and NDR thresholds in either the PFPE or PFCE experiments, providing a pool of contrast agent-agnostic features for ¹⁹F MRI (Supplementary Fig. S8). Supplementary Table S4 offers detailed lists of repeatable and stable radiomic features under different experimental conditions.

We additionally performed a sensitivity analysis varying the CCC threshold from 0.75 to 0.90 and the NDR threshold from 0.85 to 0.95. Across this grid, the qualitative conclusions remained unchanged: intensity features were the most repeatable, while shape features were the most sensitive to intersession variation. Detailed results are provided in the Supplementary Table S5.

### *In vivo* validation

**Fig. 6:**
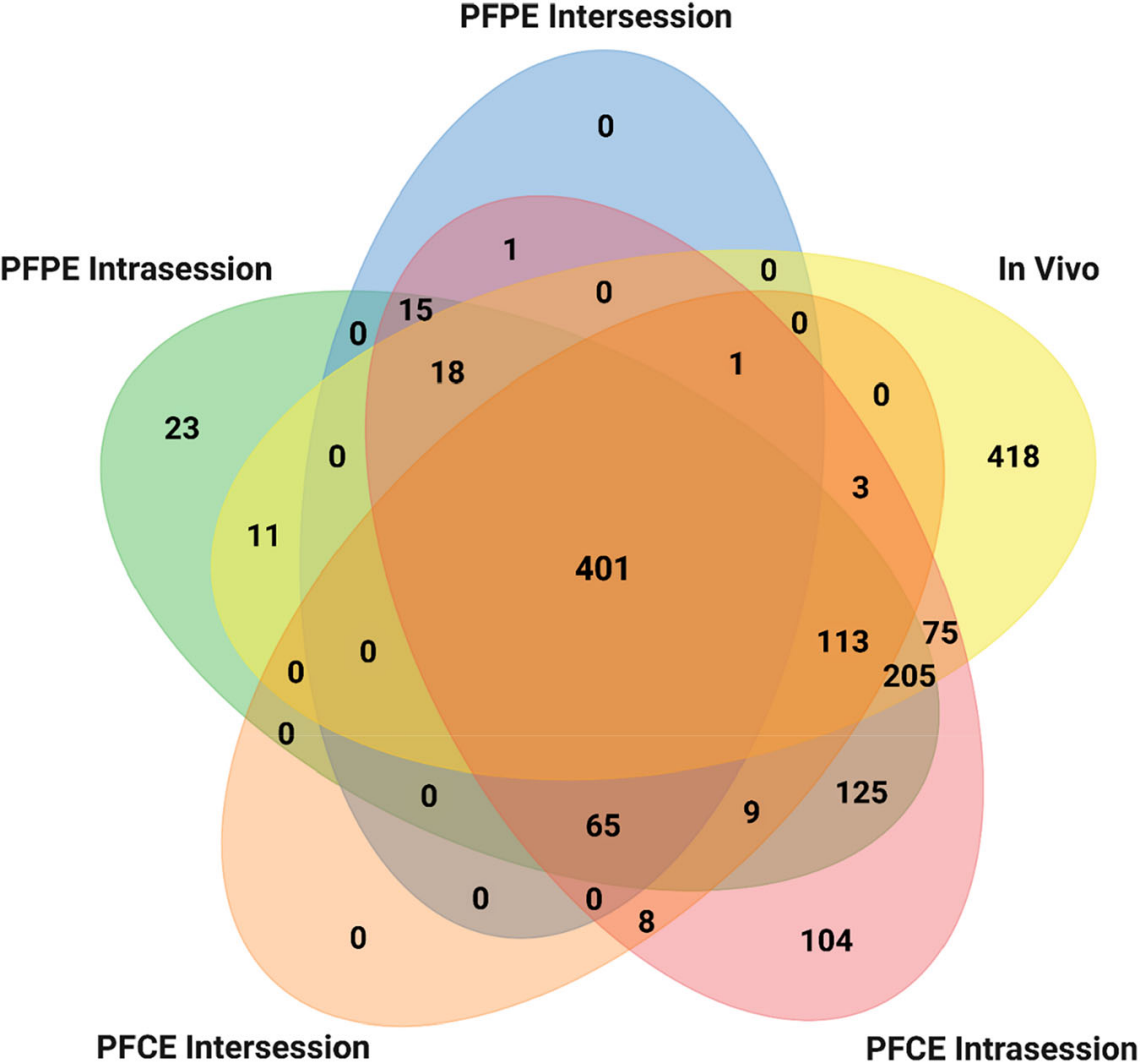
Repeatable radiomic features across different ¹⁹F MRI test-retest settings. Venn diagram illustrating the number of repeatable radiomic features shared across intra- and intersession ¹⁹F MRI test-retest trials. The trials were done in agarose phantoms containing PFPE or PFCE. An intrasession test-retest trial was done in an *in vivo* murine model of heterotopic hepatocellular carcinoma. The intersections of the ellipses represent radiomic features repeatable across multiple trials. PFCE, Perfluoro-15-crown-5 ether; PFPE, Perfluoropolyether

Having identified 466 features that remained stable across both PFPE and PFCE phantoms, we next evaluated their reliability under physiological conditions, which often introduce additional variability. Therefore, we conducted *in vivo* experiments in mice inoculated with subcutaneous liver cancer tumors in the flank. These mice received an intravenous injection of a PFPE nanoemulsion and underwent an intrasession test-retest ¹⁹F MRI 2 days after the injection (Fig. 2g–i). In total, 1,245 of 2,013 radiomic features (61.8%) exceeded the stability thresholds during these *in vivo* scans (Fig. 5g, h), with shape features showing the highest stability (78.6%, 11/14), followed by intensity (67.0%, 264/394) and texture (60.4%, 970/1,605). Crucially, 401 (86.1%) of the 466 phantom-stable features remained robust *in vivo* (Fig. 6). Intensity features formed 28.7% (115/401) of this stable set of radiomic features, while texture features comprised the remaining 71.3% (286/401).

## Discussion

Applying radiomics techniques to ¹⁹F MRI may unlock valuable quantitative biomarkers beyond signal-to-noise ratio and fluorine concentration [41], facilitating non-invasive monitoring of biological processes such as immunotherapy and cell-based treatments. While numerous studies on the robustness of radiomic features have been performed for various imaging modalities [42–44], this knowledge is currently lacking in ¹⁹F MRI. Our study addressed this gap by assessing the repeatability of radiomic features extracted from ¹⁹F MRI scans labeled with perfluoropolyether or perfluoro-15-crown-5 ether, and in a pilot *in vivo* mouse model. We identified a subset of features demonstrating high intrasession repeatability (> 50% of features with CCC ≥ 0.85). However, overall repeatability decreased in the intersession analysis (median CCC = 0.683, *p* < 0.001). In total, 466 features remained stable (CCC ≥ 0.85, NDR ≥ 0.90) across both perfluoropolyether and perfluoro-15-crown-5 ether phantoms in intra- and intersession scans, 86.1% (*n* = 401) of which also proved robust *in vivo*. These results suggest that stable ¹⁹F MRI radiomic features may provide reliable quantitative imaging markers across different perfluorocarbon agents and biological contexts. In particular, the radiomic features that were stable across all tests are intensity- and texture-based, indicating that intensity distributions and spatial relationships between voxels are more repeatable measures in ¹⁹F MRI than the geometric properties of a given ROI.

Furthermore, shape features, known for high repeatability [42, 45], were stable only in the intrasession trial. These features may be influenced by the low resolution and partial volume effects associated with ¹⁹F MR imaging. Within our analysis, the subset of radiomic features that remained stable across intrasession, intersession, and *in vivo* experiments constitutes what we consider potential “core” imaging biomarkers for ¹⁹F MRI radiomics. This core set predominantly comprises first-order intensity features (*e.g*., 90th percentile, mean, median, entropy, variance) alongside a diverse collection of texture features. These features were computed not only from the original images but also after applying a range of filters, including exponential, gradient, log-sigma, square, and wavelet filters, highlighting robustness even under different image transformation strategies. A substantial portion of the stable features is derived from images filtered using log-sigma at multiple sigma levels and wavelet filters with LLL decomposition (Supplementary Table S4), consistent with findings from previous MRI and computed tomography repeatability studies [46, 47]. These filters are more resilient to variability by reducing noise and smoothing the image.

While most ¹⁹F MRI studies prioritize qualitative co-localization with anatomical ¹H MRI, signal intensity quantification within defined regions of interest offers distinct advantages [5, 48]. Unlike indirect volumetric assessments, ¹⁹F intensity measurements directly reflect local fluorine concentrations while minimizing partial volume effects, a critical benefit given the modality’s typical spatial resolution constraints. As such, simple total voxel intensity provides a straightforward quantitative readout, capitalizing on the background-free property of ¹⁹F MRI to enhance sensitivity and specificity. Our findings align with these theoretical advantages: intensity features emerged as the most repeatable and stable radiomic feature class across all experiments. Notably, they constituted 28.6% of the final stable feature subset despite representing only 19.6% of the total feature pool. This robustness likely stems from two factors. First, the absence of an anatomic background in ¹⁹F MRI simplifies segmentation and reduces confounding variations often affecting ¹H MRI radiomics. Second, because the MR signal in ¹⁹F imaging correlates linearly with ¹⁹F concentration (unlike ¹H MRI), intensity-based metrics may be intrinsically more reproducible across scans and agents. Together, these results position intensity-focused parameters as foundational elements for developing reliable quantitative biomarkers in ¹⁹F MRI, particularly for applications that demand high reproducibility, such as the longitudinal monitoring of cellular therapies or inflammatory processes.

From a translational point of view, establishing a reproducible radiomic “core” in ^19^F MRI is an important requirement for using this modality as a quantitative endpoint in preclinical and early-phase clinical studies. Our phantom and *in vivo* experiments provide a basis that can be directly extended to human ^19^F MRI protocols, in which similar study designs and pre-selected stable radiomic features could be used to monitor cell-based therapies, immune activation, or inflammatory burden over time. Because the identified core features are robust across two representative perfluorocarbon agents and across repeated measurements, they might be well-suited to serve as harmonized imaging readouts in multicentre trials. Quantitative imaging in these contexts is usually challenged by feature instability and center-specific variability. In practice, these radiomic features could be incorporated into standard operating procedures for ^19^F MRI studies to define quantitative thresholds for successful cell delivery, to characterize intra- and inter-lesional tracer distribution, or to stratify subjects by spatial patterns of immune or inflammatory activity. Demonstrating repeatability at this preclinical stage is therefore a necessary step on the path toward regulatory acceptance of ^19^F radiomics as a robust quantitative tool to support decision-making in immunotherapy, regenerative medicine, and other molecular imaging applications.

Although intensity features proved the most robust in our experiments, certain texture-based features also demonstrated good stability. Importantly, the ‘texture’ measured here does not reflect microscopic material heterogeneity but macroscopic intensity non-uniformities introduced by the imaging system. Low-frequency bias fields (B1/coil profile), minor B0/shimming differences, and edge partial volume produce repeatable intrasession spatial patterns that change modestly with repositioning, which explains the observed drop in intersession texture and shape repeatability, while first-order intensity remains comparatively robust. Texture features capture subtle spatial variations in the signal, which may be particularly relevant in ¹⁹F imaging where heterogeneous tracer distribution (*e.g*., cellular uptake or nanoparticle clustering) can lead to spatially distinct patterns. Many texture metrics remained stable between scans, especially in the intrasession setting, reflecting their capacity to reliably encode local variations in ¹⁹F signal intensity. In contrast, shape features were only stable in the immediate test-retest setting; none of them satisfied the combined repeatability (CCC ≥ 0.85) and dynamic range (NDR ≥ 0.90) thresholds across both PFPE and PFCE phantoms once intersession factors were introduced. This instability likely arises from two interrelated factors: the spatial resolution of ¹⁹F MRI is often lower than ¹H MRI, making reliable measurements of volume, sphericity, or other geometric parameters challenging, particularly for small objects or regions. Furthermore, partial volume effects, where signal spillover occurs into neighboring voxels, combined with day-to-day sample positioning variations, can disproportionately impact shape features. While ¹⁹F MRI is not primarily used to quantify anatomical volumes, these findings emphasize the importance of focusing on intensity and texture features in contexts requiring intersession reproducibility.

Beyond feature type, the timing and conditions of test-retest trials, intrasession versus intersession, emerged as critical determinants of radiomic repeatability. While intrasession scans demonstrated high overall consistency, repeatability fell significantly when scans were done days apart and required repositioning. This temporal gap can introduce variations in sample orientation, B0 and B1 field homogeneity, and shimming, each of which can affect the detected ¹⁹F signal amplitude and spatial distribution [49, 50]. Chemical differences between PFCE and PFPE may have further accentuated day-to-day variability. PFCE, having a single resonance at around -92.8 ppm [51], yielded a cleaner spectral profile and slightly higher repeatability than PFPE, which is a heterogeneous mixture of polymers of various lengths, resulting in slight resonance peak variations of the major peak (-90.7 to -90.9 ppm) depending on chain length and conformation within the chemical environment [51]. At high PFPE concentrations, minor resonance peaks (*e.g*., around -93 ppm, from the end groups) could introduce slight shifts in the measured signal, potentially contributing to variability over time. These findings highlight how both hardware-related factors (field uniformity, coil matching) and agent-specific spectral properties can shape ¹⁹F radiomic reliability. Despite these challenges, identifying a substantial subset of features stable across agents, time points, and experimental conditions demonstrates the feasibility of developing robust ¹⁹F radiomic biomarkers resilient to real-world scanning variability.

Despite the extensive experimentation aimed at identifying stable ¹⁹F radiomic features, limitations in our study should be noted. Our findings derive from a single-scanner setup (Bruker 7-T system), which may limit generalizability to other field strengths and scanning platforms. We tested only two perfluorocarbons (PFPE and PFCE); other fluorinated agents or higher/lower concentrations might yield different patterns of radiomic stability. Although we included a preliminary *in vivo* mouse model, the sample size was modest. This pilot was intended to confirm feasibility and does not aim to capture the full extent of biological variability that may affect radiomic features. Future research focusing on *in vivo* investigations should account for sample size, anesthesia effects due to isoflurane, motion, and signal normalization both in pre- and post-processing. While appropriate for ¹⁹F imaging, our threshold-based segmentation strategy may not capture the variability that arises in more heterogeneous tissues or at lower SNR levels. Finally, this study focused on repeatability rather than reproducibility; multi-institutional analyses with varied scanners and imaging protocols will be required to confirm the broader applicability of these findings.

In summary, this study set out to determine whether radiomic features derived from ¹⁹F MRI are repeatable across different scan conditions and perfluorocarbon agents, and to identify a subset suitable for future quantitative applications. We showed that many features, particularly first-order intensity and a proportion of texture metrics, exhibited high intrasession repeatability, whereas intersession repeatability was lower, with shape metrics being most affected. Using prespecified stability criteria of CCC ≥ 0.85 and NDR ≥ 0.90, we identified a cross-agent set of stable features in phantoms, of which 401 out of 466 remained stable in the pilot *in vivo* study, supporting an agent-agnostic core for ¹⁹F radiomics. These findings provide a foundation for the development of quantitative biomarkers in this modality.

Next steps should extend beyond repeatability to formal reproducibility. Priorities include broader multicenter and multi-vendor evaluations across field strengths and acquisition protocols, as well as assessment at clinical field strengths and use cases. Prospective preclinical and early clinical studies should test whether the proposed core features remain stable under routine conditions and capture biologically or clinically meaningful changes over time, such as during cell tracking or immunotherapy monitoring. Developing harmonized acquisition and preprocessing pipelines, alongside shared phantom and *in vivo* benchmarks, will facilitate validation and accelerate clinical translation.

## Supplementary information

**Additional file 1:Table S1**. ¹H/¹⁹F MRI sequence parameters used for the PFPE/PFCE phantom and *in vivo* studies. **Table S2**. Shapiro–Wilk normality test results for concordance correlation coefficient (CCC) distributions across all reproducibility experiments. Each row reports the CCC type (PFPE, PFCE, or *in vivo*; intra- or intersession), feature group (all features, shape, intensity, or texture), the Shapiro–Wilk statistic (W), and the corresponding *p*-value. *p* < 0.05 indicates a significant deviation from a normal distribution. **Table S3**. Per-ROI voxel counts. Median and IQR of the number of voxels that were included in the thresholded segmentation per unique image type and segment. **Table S4**. Lists of repeatable radiomic features per test-retest trial type and threshold are provided in a .xlsx file. **Table S5**. Influence of thresholds of the number of stable features. Sensitivity of the number of stable radiomic features to CCC and NDR thresholds across experiments. **Fig. S1**. Hotspot sensitivity of 19F MRI. (**a**) Axial turboRARE 1H MRI of an agarose phantom containing six embedded capillaries filled with perfluoro-15-crown-5-ether (PFCE) at 19F concentrations ranging from 0‒400 mM (inner diameter 1.5 mm). (**b**) Axial 1H/19F MRI composite image with 1H shown in greyscale and 19F in bluescale. The threshold for 19F signal detection was set at signal-to-noise ratio (SNR) = 5. (**c**) Calibration curve (black line) of mean signal intensity versus 19F concentration (black triangles). Error bars indicate the standard deviation of three measurements. The red line marks the detection threshold, defined as five times the standard deviation of the noise. **Fig. S2**. ¹⁹F MRI test-retest scans. Representative ¹⁹F MRI test-retest scans acquired in the same session (intrasession) for PFPE (left) and PFCE (middle) phantoms and for an *in vivo* mouse model (right). The top row shows the initial “test” scans, and the bottom row displays the “retest” scans under identical imaging conditions. *PFCE* **Fig. S3.** Comparison of voxel intensity histograms between test and retest ¹⁹F MR scans acquired within the same imaging session (intrasession). This figure illustrates the non-normalized (top row) and z-score normalized (bottom row) intensity histograms for each segmented region in the test (blue) and retest (orange) scans. The Jensen–Shannon Divergence is displayed in each subplot to quantify the similarity of intensity distributions for intrasession measurements. **Fig. S4**. Comparison of voxel intensity distributions between test and retest scans acquired in imaging sessions separated by time (intersession). Shown here are the non-normalized (top row) and (z-score) normalized (bottom row) histograms of voxel intensities for each segmented region from the test (blue) and retest (orange) scans. The Jensen–Shannon Divergence values in each subplot measure how closely the test and retest intensity distributions match in this intersession scenario. **Fig. S5**. Distribution of the concordance correlation coefficient of radiomic features stratified per filter class. Violin plots representing the CCC distribution for different radiomic filter classes in the PFPE intrasession trial. The red line and black lines represent the median and quartiles, respectively. **Fig. S6**. Assessment of particle sedimentation in two agarose phantoms containing 19F–labeled nanocarriers. Each 1.5-mL tube was filled with 1% (w/v) low-melting-point agarose (in PBS) infused with either PFCE nanoparticles or a PFPE nanoemulsion and scanned longitudinally by ¹⁹F MRI using a fast spin-echo sequence. The center panel (¹H localizer) illustrates the sagittal slice geometry, with colored lines denoting three distinct regions for analysis: top (red/orange), center (yellow/green), and bottom (blue/purple). A region of interest was drawn in each slice to calculate the SNR using Rician noise correction. Plots to the left and right show the temporal SNR measurements in the PFCE and PFPE phantoms, respectively, highlighting changes in different tube regions over time. **Fig. S7**. Bland-Altman plots with concordance correlation coefficient (CCC) of individual 19F MRI radiomic features across intrasession and intersession test-retest trials of an agarose phantom containing PFPE or PFCE. The mean value of a radiomic feature (horizontal axis) is plotted against the difference between the test and retest (vertical axis). The 95% limits of agreement are represented by the green dashed lines. The red line depicts the mean of the differences between test-retest, indicating general bias. A reference band is shown (blue dash-dot line), calculated according to Kim and Lee [38]. The reference band represents the expected range of differences if the target CCC = 0.85. For each plot, the provided CCC is the product of the Pearson correlation coefficient (ρ) and the bias correction factor (Cb). **Fig. S8**. Overlapping results of a repeatable radiomic feature signature extracted from 7-T ¹⁹F MRI scans performed in two different agarose-perfluorocarbon phantoms. Both phantoms containing either PFPE or PFCE were scanned twice within a single session with no repositioning (intrasession). The intersession test-retest trial included an additional scan performed five to seven days later with repositioning of the phantom. The intersections of the ellipses represent radiomic features, which were repeatable across multiple trials.

## Data Availability

The datasets generated and/or analyzed during the current study are not publicly available due to institutional data sharing policies, but are available from the corresponding author on reasonable request.

## Abbreviations

3D: Three-dimensional
CCC: Concordance correlation coefficient
IQR: Interquartile range
MRI: Magnetic resonance imaging
N_average_: Average noise level
NDR: Normalized dynamic range
PFCE: Perfluoro-15-crown-5 ether
PFPE: Perfluoropolyether
RARE: Rapid acquisition with relaxation enhancement
ROI: Region of interest
SNR: Signal-to-noise ratio

## Code availability

The code used for data processing and statistical analyses is available at the following GitHub repository: https://github.com/nki-radiology/19F_radiomics_repeatability.
