# Supplementary material for: ¹⁹F MRI radiomic features: in vitro and in vivo repeatability
Source: Eur Radiol Exp. 2026 Mar 16;10:29. doi: 10.1186/s41747-026-00694-2 (PMC12992713; doi:10.1186/s41747-026-00694-2)
Supplement: Supplementary file 1 — Additional file 1:Table S1. ¹H/¹⁹F MRI sequence parameters used for the PFPE/PFCE phantom and in vivo studies. Table S2. Shapiro–Wilk normality test results for concordance correlation coefficient (CCC) distributions across all reproducibility experiments. Each row reports the CCC type (PFPE, PFCE, or in vivo; intra- or intersession), feature group (all features, shape, intensity, or texture), the Shapiro–Wilk statistic (W), and the corresponding p-value. p < 0.05 indicates a significant deviation from a normal distribution. Table S3. Per-ROI voxel counts. Median and IQR of the number of voxels that were included in the thresholded segmentation per unique image type and segment. Table S4. Lists of repeatable radiomic features per test-retest trial type and threshold are provided in a .xlsx file. Table S5. Influence of thresholds of the number of stable features. Sensitivity of the number of stable radiomic features to CCC and NDR thresholds across experiments. Fig. S1. Hotspot sensitivity of 19F MRI. (a) Axial turboRARE 1H MRI of an agarose phantom containing six embedded capillaries filled with perfluoro-15-crown-5-ether (PFCE) at 19F concentrations ranging from 0‒400 mM (inner diameter 1.5 mm). (b) Axial 1H/19F MRI composite image with 1H shown in greyscale and 19F in bluescale. The threshold for 19F signal detection was set at signal-to-noise ratio (SNR) = 5. (c) Calibration curve (black line) of mean signal intensity versus 19F concentration (black triangles). Error bars indicate the standard deviation of three measurements. The red line marks the detection threshold, defined as five times the standard deviation of the noise. Fig. S2. ¹⁹F MRI test-retest scans. Representative ¹⁹F MRI test-retest scans acquired in the same session (intrasession) for PFPE (left) and PFCE (middle) phantoms and for an in vivo mouse model (right). The top row shows the initial “test” scans, and the bottom row displays the “retest” scans under identical imaging conditions. PFCE Fig. S [file 41747_2026_694_MOESM1_ESM.pdf]

^{19}F MRI radiomic features: in vitro and in vivo repeatability

ELECTRONIC SUPPLEMENTARY MATERIAL

Supplementary Methods Section 1

Animal husbandry

Two female C57BL/6 mice (6 weeks old; Janvier Labs, France) were used in this study. The mice were housed in individually ventilated cages (Innovive Disposable IVC Rodent Caging System, San Diego, CA, USA) at the Netherlands Cancer Institute animal laboratory facility. They were maintained under specific pathogen-free conditions with a 12-hour light/dark cycle and had *ad libitum* access to food and water. Cage bedding was changed weekly, and animal health and welfare were monitored twice a week; any concerning observations were addressed daily.

Cell line and in vivo model

Primary hepatocellular carcinoma cells were isolated from a somatic mouse model induced by hydrodynamic tail vein injection, which carried Myc overexpression along with p53 knockout ($Myc^{OE}/p53^{KO}$), as described in prior studies [31, 50, 51]. Tumor nodules were macrodissected from the liver and dissociated into a single-cell suspension. $Myc^{OE}/p53^{KO}$ cancer cells were then seeded onto Collagen Type I rat tail pre-coated dishes (Corning, cat.no. 354236) and grown in Dulbecco's Modified Eagle Medium supplemented with 10% fetal bovine serum and maintained at 37 °C and in a humidified atmosphere with 5% CO2. For the heterotopic HCC model, subcutaneous tumors were established by injecting $2.5*10^4$ $Myc^{OE}/p53^{KO}$ hepatocellular carcinoma cells suspended in Matrigel (Corning, cat.no. 356230) into the right flank of each mouse (n=2). Tumor growth was monitored twice a week using calliper measurements. When tumors reached a palpable size (>100 mm^3), mice received an intravenous injection of 200 µl of PFPE nanoemulsion (120 mg PFPE/ml, VS-1000H DM Red; Celsense Inc., Pittsburgh, PA, USA) via the tail vein to enable ^{19}F MRI.

Supplementary Methods Section 2. Pyradiomics parameter settings for radiomics feature extraction.

```
imageType:

  Original:
    binWidth: 6.0
  Square:
    binWidth: 3.9
  SquareRoot:
    binWidth: 6.2
  Logarithm:
    binWidth: 4.0
  Exponential:
    binWidth: 0.7
  Gradient:
    binWidth: 4.0
  LoG:
    binWidth: 2.5
    sigma: [1.0, 2.0, 3.0, 4.0, 5.0]
  Wavelet:
    binWidth: 5.0
  LBP3D:
    binWidth: 1.0

featureClass:
  shape:
  firstorder:
  glcm:
    - 'Autocorrelation'
    - 'JointAverage'
    - 'ClusterProminence'
    - 'ClusterShade'
    - 'ClusterTendency'
    - 'Contrast'
    - 'Correlation'
    - 'DifferenceAverage'
    - 'DifferenceEntropy'
    - 'DifferenceVariance'
```

    - 'JointEnergy'
    - 'JointEntropy'
    - 'Imc1'
    - 'Imc2'
    - 'Idm'
    - 'Idmn'
    - 'Id'
    - 'Idn'
    - 'InverseVariance'
    - 'MaximumProbability'
    - 'SumEntropy'
    - 'SumSquares'
  glrlm:
  glszm:
  gldm:
  ngtdm:

setting:
  interpolator: 'sitkBSpline'
  resampledPixelSpacing: [1, 1, 1]
  padDistance: 10

  minimumROIDimensions: 2
  minimumROISize: 1
  correctMask: true

  voxelArrayShift: 1000

 label: 1

Supplementary Methods Section 3. List of excluded features.

lbp-3D-k_ngtdm_Strength
lbp-3D-m2_firstorder_Minimum
lbp-3D-m1_firstorder_Minimum

Supplementary Methods Section 4. Description of intra-reader and inter-reader variability calculation.

Intrareader variability assessment

To assess the reproducibility of radiomic features based on intra-reader segmentation variability, we randomly selected 20 ^{19}F MRI scans from our PFPE phantom cohort. A blinded observer performed two independent segmentations on each scan. The segmentation method was identical to the approach described in the main Methods section: (1) a mask was generated to include only voxels with an intensity ≥three times the $N_{average}$, and (2) the region of interest for each phantom tube was manually delineated. Radiomic features were then extracted from each segment. The intra-class correlation coefficient (ICC) was calculated for each radiomic feature to evaluate reproducibility. We employed a two-way mixed-effects model (ICC 3,1) to assess single-rater absolute agreement. The ICC values, 95% confidence intervals, and p-values were computed using the *pingouin* Python package (v0.5.3). We reported the ICC results as the median ICC value, accompanied by the interquartile range (IQR, 25th–75th percentile).

Inter-reader variability assessment

To evaluate the reproducibility of radiomic features across different segmenters, we assessed inter-reader variability in the segmentation process. Two independent observers (O.M., D.I.R.S.) each segmented 40 ^{19}F MRI scans, comprising 20 PFPE and 20 PFCE phantoms, using the same segmentation procedure described in the main Methods section: (1) a mask was generated to include only voxels with an intensity ≥ three times the $N_{average}$, and (2) the region of interest for each phantom tube was manually delineated. Radiomic features were extracted from each set of segmentations. Inter-reader agreement was assessed by calculating the ICC for each feature using a two-way random-effects model (ICC 2,1), which quantifies inter-rater absolute agreement. ICC values, 95% confidence intervals, and p-values were calculated using the *pingouin* Python package (v0.5.3). We summarised reproducibility using the median ICC across all features, along with the interquartile range (IQR, 25th–75th percentile).

Supplementary Methods Section 5. Description of the approach to determine the sedimentation rate of the perfluorocarbons within the PFPE/PFCE phantoms.

Perfluorocarbon sedimentation rate within phantoms

To evaluate potential sedimentation of PFPE nanoemulsions and PFCE nanoparticles within agarose phantoms, a longitudinal ^{19}F MRI study was conducted. Two separate 1.5 ml tube phantoms were prepared: one containing PFPE nanoparticles at 4.8 mM (201.6 mM ^{19}F content) and the other containing PFCE nanoparticles at 12 mM (240 mM ^{19}F content), each suspended in 1% (w/v) low-melting-point agarose (dissolved in phosphate-buffered saline). The phantoms were stored upright at 4 °C between imaging sessions to mimic intersession storage conditions.

Longitudinal ^{19}F MRI scans were conducted using a fast spin echo sequence with repetition time (TR) = 2,000 ms, echo time (TE) = 15 ms, echo train length = 8, matrix = 64 × 64, field of view (FOV) = 25 × 25 mm^2, slice thickness = 2 mm, and a slice gap of 0.1 mm. For the PFPE phantom, 100 excitations were acquired on days 3, 7, 10, 14, and 17. For the PFCE phantom, 150 excitations were acquired on days 4, 8, 11, 15, and 19.

In each scan, regions of interest were manually drawn on axial slices corresponding to the top (slices 12–13), center (slices 7–8), and bottom (slices 3–4) of the tube. Noise levels were quantified by measuring the standard deviation of signal intensity in four corner regions of the image. The SNR was calculated by dividing the mean pixel value of the signal ROI by the noise levels, using the Rayleigh correction factor of 0.66 to obtain the true SNR.

By comparing the SNR in each zone over successive time points, any systematic particle sedimentation could be detected in the PFPE and PFCE phantoms.

Table S1. $^{1}H/^{19}F$ MRI sequence parameters used for the PFPE/PFCE phantom and *in vivo* studies

Nucleus	Repetition time (ms)	Echo time (ms)	Turbo factor	FOV (mm)	Acquisition matrix	Slice thickness (mm)	Bandwidth (kHz)	Number of excitations	Time of acquisition
^{1}H	2500	36	8	35*35	256*256	1.5	72	2	2m 40s
^{19}F	2000	22.5	8	35*35	64*64	1.5	20	120	32m

Table S2. Shapiro–Wilk normality test results for concordance correlation coefficient (CCC) distributions across all reproducibility experiments. Each row reports the CCC type (PFPE, PFCE, or in vivo; intra- or inter-session), feature group (all features, shape, intensity, or texture), the Shapiro–Wilk statistic (W), and the corresponding p-value. p<0.05 indicates a significant deviation from a normal distribution

CCC Type	Group	Shapiro-W	*p*-value
PFPE intrasession CCC	All	0.817	< 0.001
PFPE intrasession CCC	Shape	0.895	0.096
PFPE intrasession CCC	Intensity	0.720	< 0.001
PFPE intrasession CCC	Texture	0.835	< 0.001
PFPE intersession CCC	All	0.919	< 0.001
PFPE intersession CCC	Shape	0.847	0.020
PFPE intersession CCC	Intensity	0.889	< 0.001
PFPE intersession CCC	Texture	0.923	< 0.001
PFCE intrasession CCC	All	0.825	< 0.001
PFCE intrasession CCC	Shape	0.945	0.483
PFCE intrasession CCC	Intensity	0.719	< 0.001
PFCE intrasession CCC	Texture	0.847	< 0.001
PFCE intersession CCC	All	0.911	< 0.001
PFCE intersession CCC	Shape	0.859	0.030
PFCE intersession CCC	Intensity	0.863	< 0.001
PFCE intersession	Texture	0.918	< 0.001

CCC			
In vivo intrasession CCC	All	0.645	< 0.001
In vivo intrasession CCC	Shape	0.565	< 0.001
In vivo intrasession CCC	Intensit y	0.563	< 0.001
In vivo intrasession CCC	Texture	0.674	< 0.001

Table S3. Per-ROI voxel counts. Median and IQR of the number of voxels that were included in the thresholded segmentation per unique image type and segment

	Segment name	Number of voxels
PFPE phantom	High 19F tube #1	486.0 (465.0–502.8)
	High 19F tube #2	530.5 (520.2–541.5)
	Low 19F tube #1	506.0 (493.0–518.5)
	Low 19F tube #2	446.0 (428.5–456.5)
PFCE phantom	High 19F tube #1	320.0 (315.5–324.2)
	High 19F tube #2	349.0 (342.5–356.0)
	Low 19F tube #1	332.0 (324.0–337.0)
	Low 19F tube #2	274.5 (267.8–286.2)
Mouse #1	Reference tube	386.0 (385.5–386.5)
	Liver	966.0 (953.5–978.5)
	Tumor	155.0 (152.0–158.0)
Mouse #2	Reference tube	381.5 (378.8–384.2)
	Liver	877.0 (874.0–880.0)
	Tumor	57.0 (57.0–57.0)

Table S4. Lists of repeatable radiomic features per test-retest trial type and threshold are provided in a .xlsx file

Table S5. Influence of thresholds of the number of stable features. Sensitivity of the number of stable radiomic features to CCC and NDR thresholds across experiments

CCC threshold	NDR threshold	Scenario	Stable shape features	Stable first order features	Stable texture features	Total stable features
0.75	0.85	PFPE intrasession	10	305	1,006	1,321
0.75	0.85	PFPE intersession	1	188	655	844
0.75	0.85	PFCE intrasession	13	324	1,044	1,381
0.75	0.85	PFCE intersession	5	229	715	949
0.75	0.85	*In vivo*	13	334	1,294	1,641
0.75	0.9	PFPE intrasession	8	261	769	1,038
0.75	0.9	PFPE intersession	0	129	392	521
0.75	0.9	PFCE intrasession	13	308	956	1,277
0.75	0.9	PFCE intersession	3	185	511	699
0.75	0.9	*In vivo*	11	264	984	1,259
0.75	0.95	PFPE intrasession	1	124	312	437
0.75	0.95	PFPE intersession	0	43	36	79
0.75	0.95	PFCE intrasession	6	219	509	734
0.75	0.95	PFCE intersession	0	34	39	73
0.75	0.95	*In vivo*	7	164	468	639
0.8	0.85	PFPE intrasession	8	292	927	1,227
0.8	0.85	PFPE intersession	0	173	566	739
0.8	0.85	PFCE intrasession	11	311	963	1,285
0.8	0.85	PFCE intersession	2	201	612	815
0.8	0.85	*In vivo*	13	327	1239	1,579
0.8	0.9	PFPE intrasession	8	260	759	1,027
0.8	0.9	PFPE intersession	0	128	387	515
0.8	0.9	PFCE intrasession	11	303	924	1,238
0.8	0.9	PFCE intersession	2	182	497	681
0.8	0.9	*In vivo*	11	264	980	1,255
0.8	0.95	PFPE intrasession	1	124	312	437
0.8	0.95	PFPE intersession	0	43	36	79
0.8	0.95	PFCE intrasession	5	219	507	731
0.8	0.95	PFCE intersession	0	34	39	73
0.8	0.95	*In vivo*	7	164	468	639
0.85	0.85	PFPE intrasession	7	259	789	1,055
0.85	0.85	PFPE intersession	0	147	469	616
0.85	0.85	PFCE intrasession	11	284	853	1,148
0.85	0.85	PFCE intersession	0	151	502	653
0.85	0.85	*In vivo*	13	306	1162	1,481
0.85	0.9	PFPE intrasession	7	248	730	985
0.85	0.9	PFPE intersession	0	122	379	501
0.85	0.9	PFCE intrasession	11	282	850	1,143
0.85	0.9	PFCE intersession	0	145	455	600

0.85	0.9	*In vivo*	11	264	970	1,245
0.85	0.95	PFPE intrasession	1	124	312	437
0.85	0.95	PFPE intersession	0	43	36	79
0.85	0.95	PFCE intrasession	5	216	501	722
0.85	0.95	PFCE intersession	0	34	37	71
0.85	0.95	*In vivo*	7	164	468	639
0.9	0.85	PFPE intrasession	3	211	627	841
0.9	0.85	PFPE intersession	0	126	350	476
0.9	0.85	PFCE intrasession	6	245	685	936
0.9	0.85	PFCE intersession	0	130	379	509
0.9	0.85	*In vivo*	12	287	1024	1,323
0.9	0.9	PFPE intrasession	3	206	618	827
0.9	0.9	PFPE intersession	0	120	340	460
0.9	0.9	PFCE intrasession	6	245	685	936
0.9	0.9	PFCE intersession	0	129	375	504
0.9	0.9	*In vivo*	11	262	932	1205
0.9	0.95	PFPE intrasession	1	124	310	435
0.9	0.95	PFPE intersession	0	43	36	79
0.9	0.95	PFCE intrasession	3	204	483	690
0.9	0.95	PFCE intersession	0	34	37	71
0.9	0.95	*In vivo*	7	164	468	639

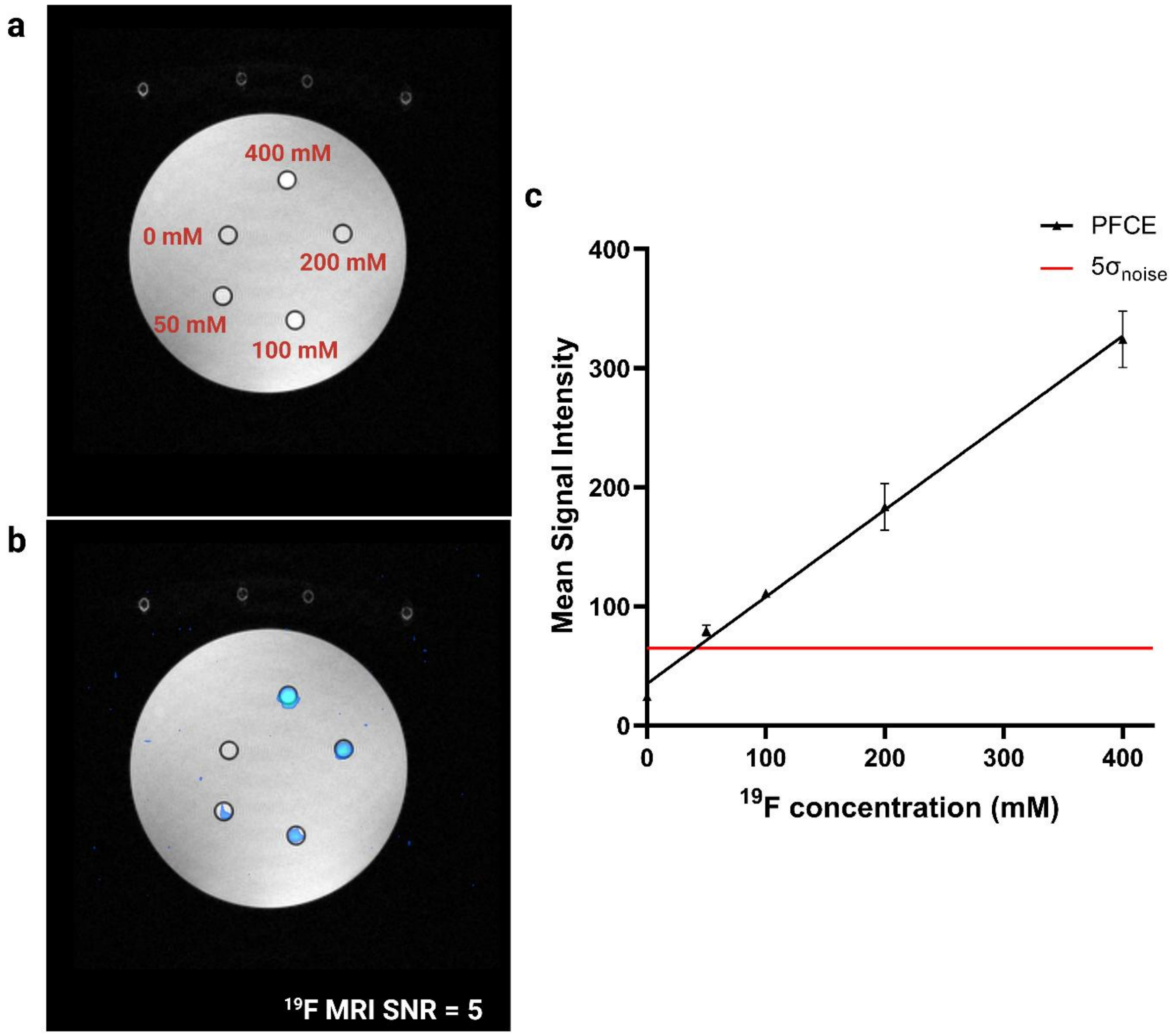

Fig. S1. Hotspot sensitivity of ^{19}F MRI. (**a**) Axial turboRARE ^{1}H MRI of an agarose phantom containing six embedded capillaries filled with perfluoro-15-crown-5-ether (PFCE) at ^{19}F concentrations ranging from 0–400 mM (inner diameter 1.5 mm). (**b**) Axial ^{1}H/^{19}F MRI composite image with ^{1}H shown in greyscale and ^{19}F in bluescale. The threshold for ^{19}F signal detection was set at signal-to-noise ratio (SNR) = 5. (**c**) Calibration curve (black line) of mean signal intensity versus ^{19}F concentration (black triangles). Error bars indicate the standard deviation of three measurements. The red line marks the detection threshold, defined as five times the standard deviation of the noise.

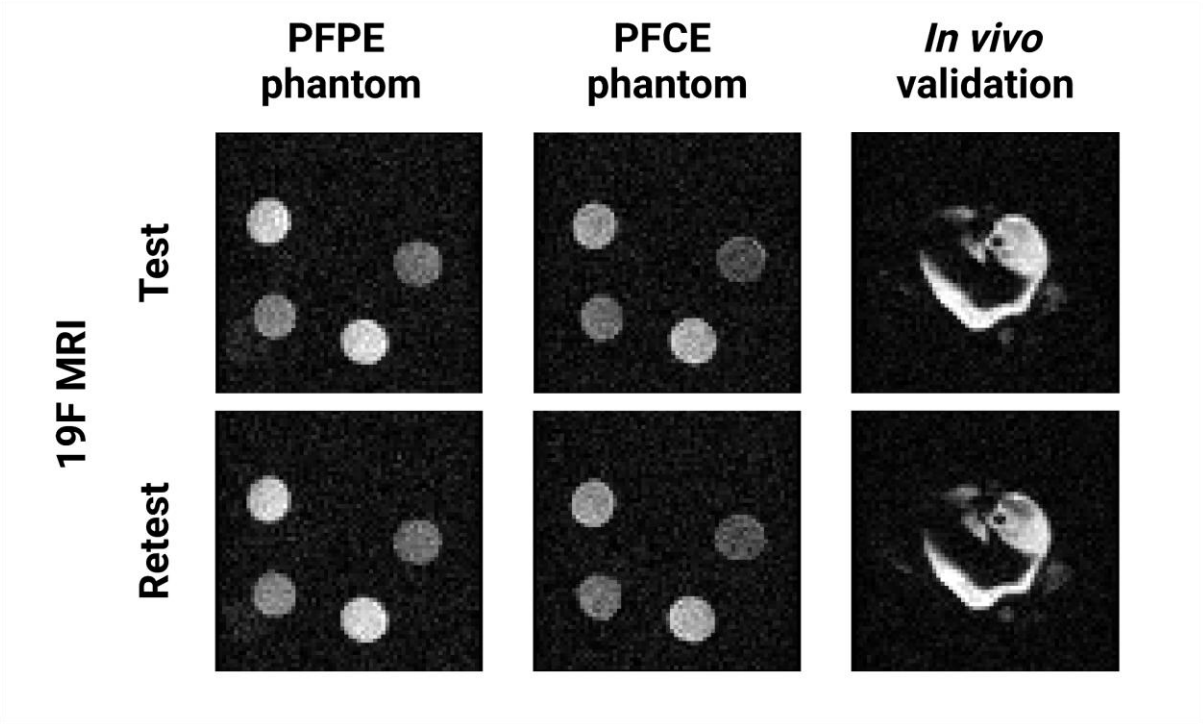

Fig. S2. ^{19}F MRI test-retest scans. Representative ^{19}F MRI test-retest scans acquired in the same session (intrasession) for PFPE (left) and PFCE (middle) phantoms and for an *in vivo* mouse model (right). The top row shows the initial “test” scans, and the bottom row displays the “retest” scans under identical imaging conditions. *PFCE* Perfluoro-15-crown-5 ether, *PFPE* Perfluoropolyether.

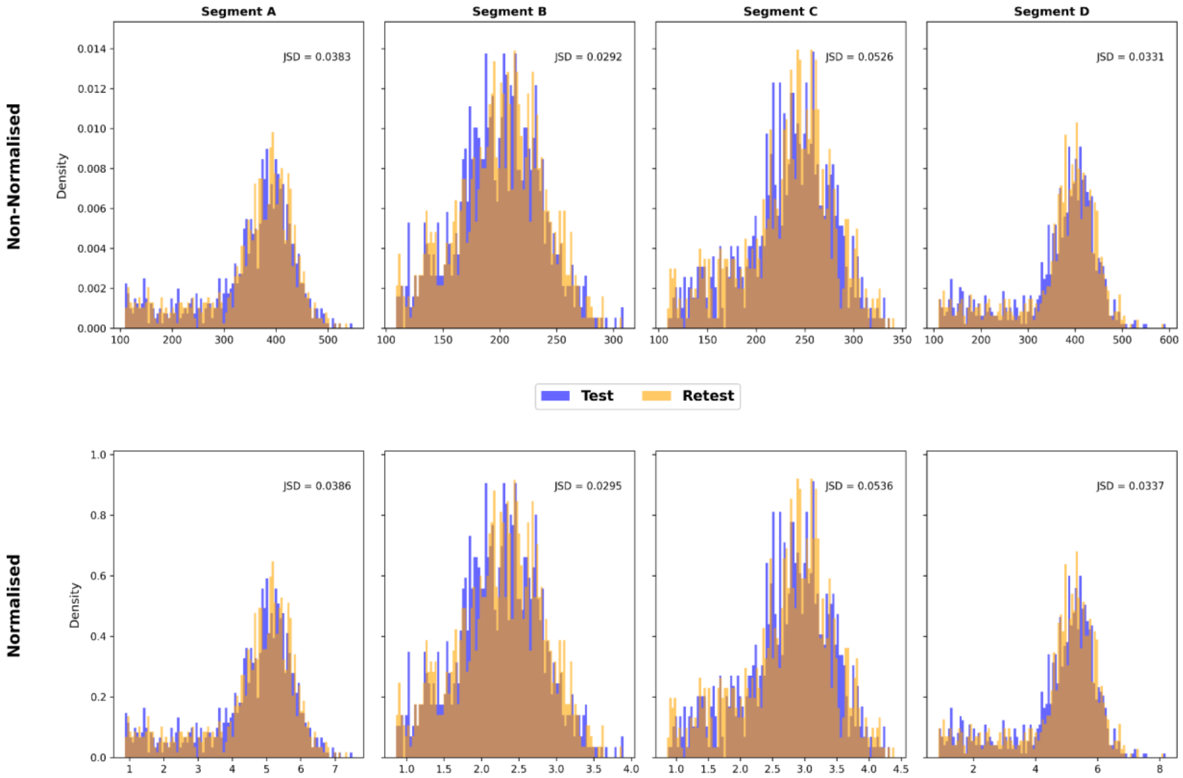

Fig. S3. Comparison of voxel intensity histograms between test and retest ^{19}F MR scans acquired within the same imaging session (intrasession). This figure illustrates the non-normalized (top row) and z-score normalized (bottom row) intensity histograms for each segmented region in the test (blue) and retest (orange) scans. The Jensen–Shannon Divergence is displayed in each subplot to quantify the similarity of intensity distributions for intrasession measurements.

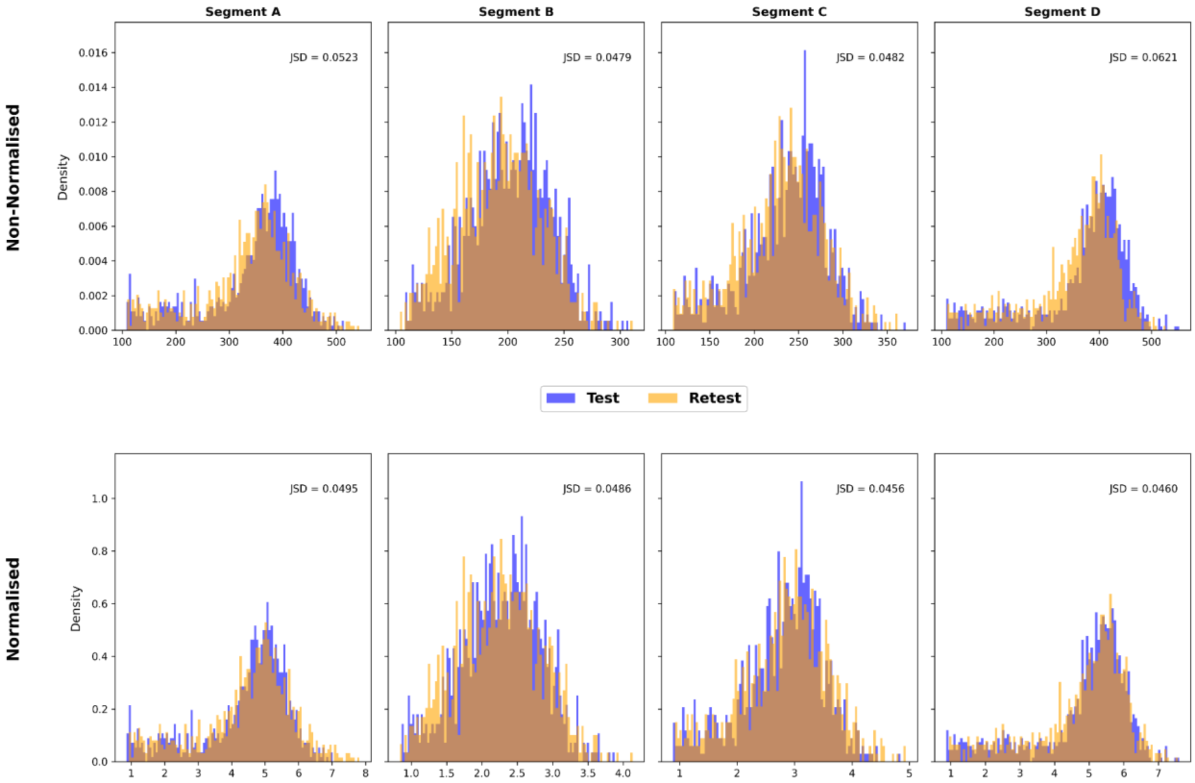

Fig. S4. Comparison of voxel intensity distributions between test and retest scans acquired in imaging sessions separated by time (intersession). Shown here are the non-normalized (top row) and (z-score) normalized (bottom row) histograms of voxel intensities for each segmented region from the test (blue) and retest (orange) scans. The Jensen–Shannon Divergence values in each subplot measure how closely the test and retest intensity distributions match in this intersession scenario.

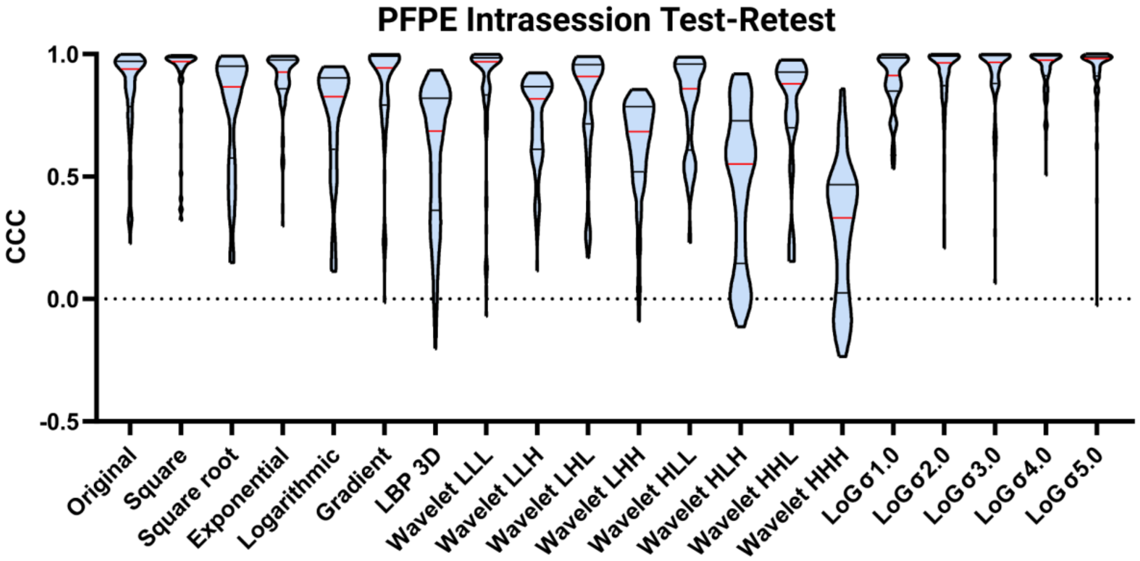

Fig. S5. Distribution of the concordance correlation coefficient of radiomic features stratified per filter class. Violin plots representing the CCC distribution for different radiomic filter classes in the PFPE intrasession trial. The red line and black lines represent the median and quartiles, respectively. CCC Concordance correlation coefficient, *LBP 3D* Local Binary Pattern in 3D, *LoG* Laplacian of Gaussian, *PFPE* Perfluoropolyether.

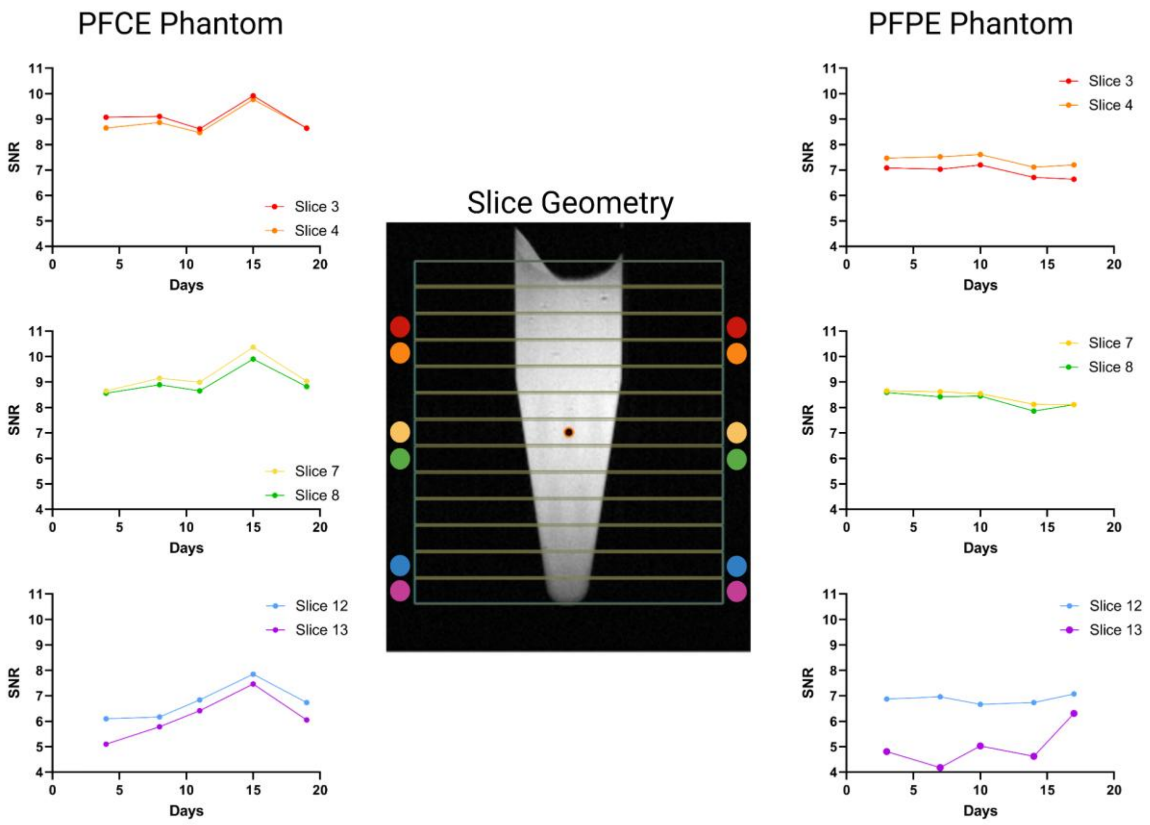

Fig. S6. Assessment of particle sedimentation in two agarose phantoms containing ^{19}F–labelled nanocarriers. Each 1.5 mL tube was filled with 1% (w/v) low-melting-point agarose (in PBS) infused with either PFCE nanoparticles or a PFPE nanoemulsion and scanned longitudinally by ^{19}F MRI using a fast spin echo sequence. The center panel (^{1}H localizer) illustrates the sagittal slice geometry, with coloured lines denoting three distinct regions for analysis: top (red/orange), center (yellow/green), and bottom (blue/purple). A region of interest was drawn in each slice to calculate the SNR using Rician noise correction. Plots to the left and right show the temporal SNR measurements in the PFCE and PFPE phantoms, respectively, highlighting changes in different tube regions over time. *PFCE* Perfluoro-15-crown-5 ether, *PFPE* Perfluoropolyether, *SNR* Signal-to-noise ratio.

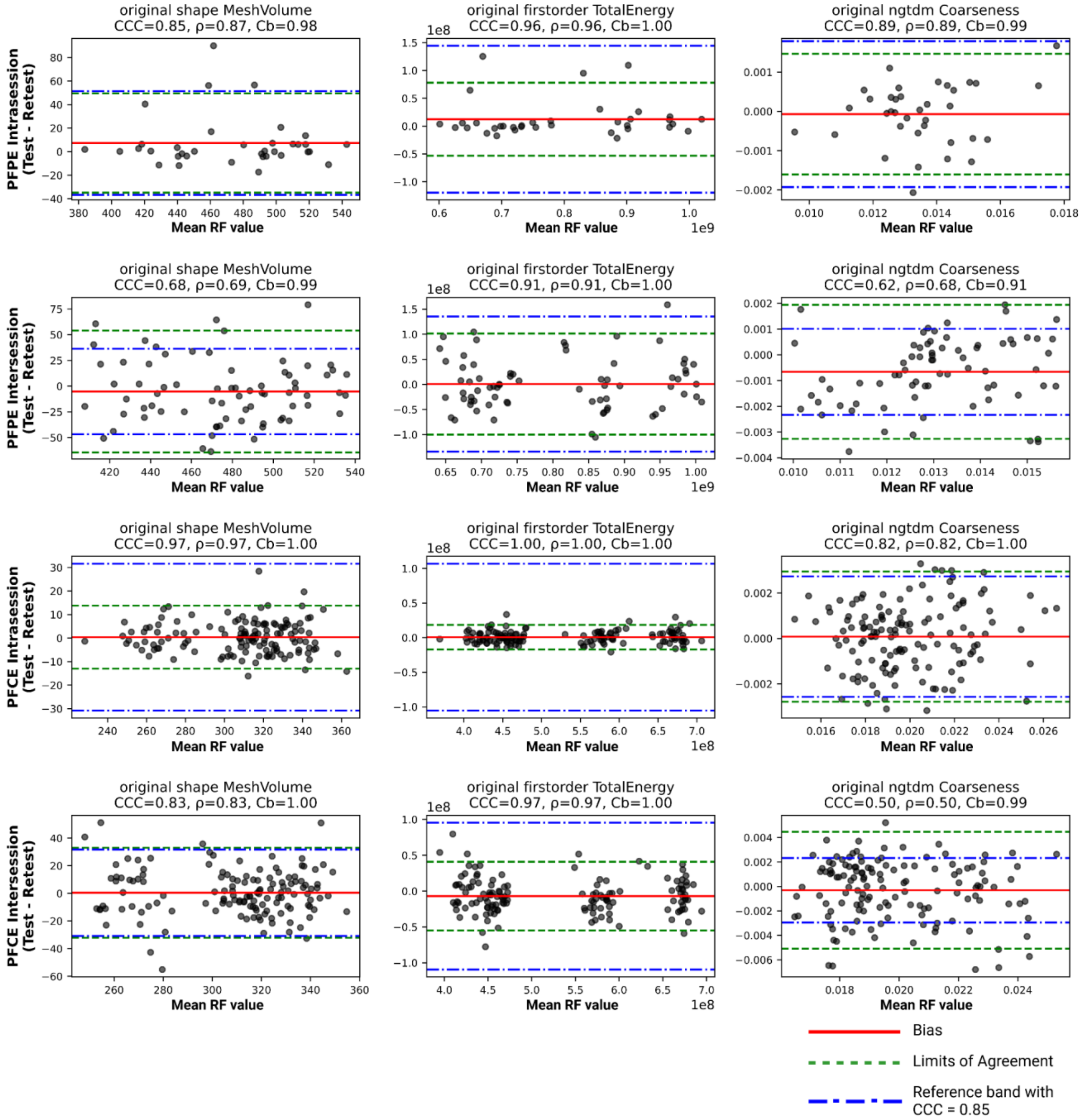

Fig. S7. Bland-Altman plots with concordance correlation coefficient (CCC) of individual 19F MRI radiomic features across intrasession and intersession test-retest trials of an agarose phantom containing PFPE or PFCE. The mean value of a radiomic feature (horizontal axis) is plotted against the difference between the test and retest (vertical axis). The 95% limits of agreement are represented by the green dashed lines. The red line depicts the mean of the differences between test-retest, indicating general bias. A reference band is shown (blue dash-dot line), calculated according to Kim and Lee [38]. The reference band represents the expected range of differences if the target CCC = 0.85. For each plot, the provided CCC is the product of the Pearson correlation coefficient (ρ) and the bias correction factor (Cb). *PFCE* Perfluoro-15-crown-5 ether, *PFP* Perfluoropolyether.

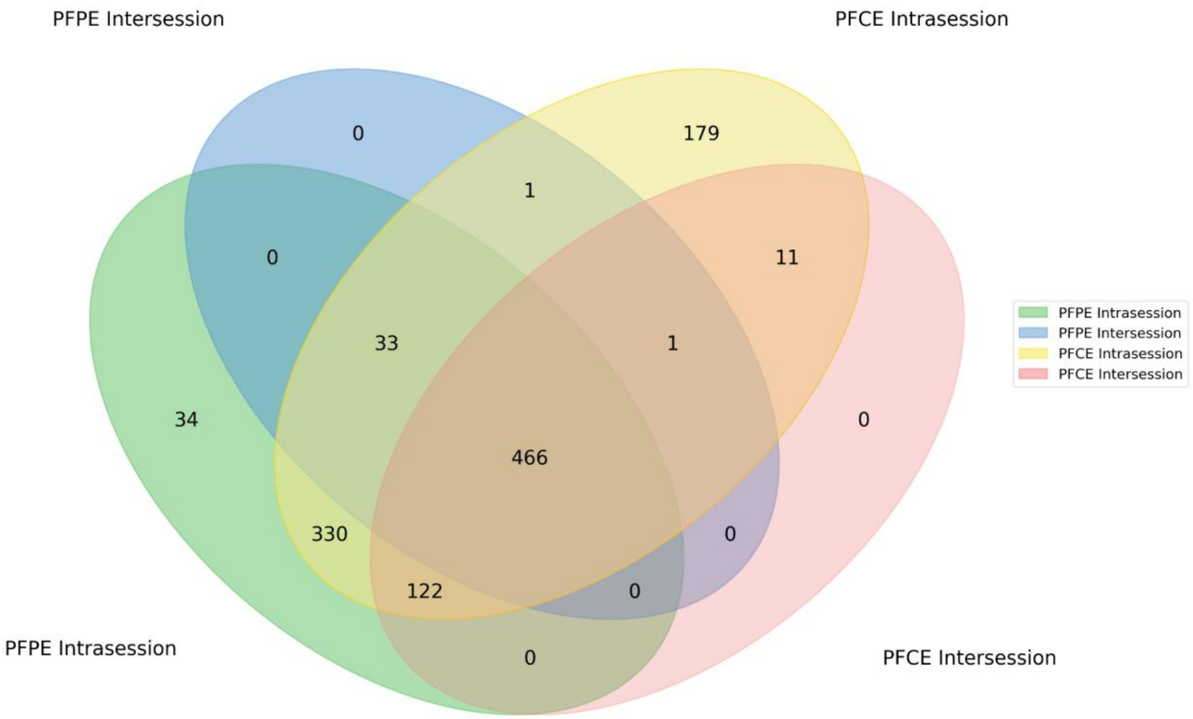

Fig. S8. Overlapping results of a repeatable radiomic feature signature extracted from 7-T ^{19}F MRI scans performed in two different agarose-perfluorocarbon phantoms. Both phantoms containing either PFPE or PFCE were scanned twice within a single session with no repositioning (intrasession). The intersession test-retest trial included an additional scan performed five to seven days later with repositioning of the phantom. The intersections of the ellipses represent radiomic features, which were repeatable across multiple trials. *PFCE* Perfluoro-15-crown-5 ether, *PFPE* Perfluoropolyether.
